# Toward Modeling Context-Specific EMT Regulatory Networks Using Temporal Single Cell RNA-Seq Data

**DOI:** 10.3389/fmolb.2020.00054

**Published:** 2020-04-23

**Authors:** Daniel Ramirez, Vivek Kohar, Mingyang Lu

**Affiliations:** ^1^College of Health Solutions, Arizona State University, Tempe, AZ, United States; ^2^The Jackson Laboratory for Mammalian Genetics, Bar Harbor, ME, United States

**Keywords:** phenotypic plasticity, epithelial-mesenchymal transition, cancer, network modeling, single-cell RNA-seq

## Abstract

Epithelial-mesenchymal transition (EMT) is well established as playing a crucial role in cancer progression and being a potential therapeutic target. To elucidate the gene regulation that drives the decision making of EMT, many previous studies have been conducted to model EMT gene regulatory circuits (GRCs) using interactions from the literature. While this approach can depict the generic regulatory interactions, it falls short of capturing context-specific features. Here, we explore the effectiveness of a combined bioinformatics and mathematical modeling approach to construct context-specific EMT GRCs directly from transcriptomics data. Using time-series single cell RNA-sequencing data from four different cancer cell lines treated with three EMT-inducing signals, we identify context-specific activity dynamics of common EMT transcription factors. In particular, we observe distinct paths during the forward and backward transitions, as is evident from the dynamics of major regulators such as NF-KB (e.g., NFKB2 and RELB) and AP-1 (e.g., FOSL1 and JUNB). For each experimental condition, we systematically sample a large set of network models and identify the optimal GRC capturing context-specific EMT states using a mathematical modeling method named *Ra*ndom *Ci*rcuit *Pe*rturbation (RACIPE). The results demonstrate that the approach can build high quality GRCs in certain cases, but not others and, meanwhile, elucidate the role of common bioinformatics parameters and properties of network structures in determining the quality of GRCs. We expect the integration of top-down bioinformatics and bottom-up systems biology modeling to be a powerful and generally applicable approach to elucidate gene regulatory mechanisms of cellular state transitions.

## Introduction

Epithelial-mesenchymal transition (EMT) has been implicated in a number of biological phenomena including embryonic development, wound healing, and cancer metastasis (Thiery et al., 2009). During EMT, epithelial cells detach from their environment and gain more migratory and apoptosis-resistant qualities (Nieto et al., 2016) to become mesenchymal cells (Nistico et al., 2012). Recent studies have identified new hybrid EMT cellular states (Bartoschek et al., 2018; Dong et al., 2018) with the expression of both epithelial (E) and mesenchymal (M) genes. The hybrid states in cancer have been associated with collective cell migration and aggressiveness of cancer (Jolly, 2015).

From extensive experimental (Ding et al., 2013; Bartoschek et al., 2018; Dong et al., 2018) and computational (Steinway et al., 2014; Burger et al., 2017; Jia et al., 2019) studies, it is now understood that the decision making of an EMT is usually driven by a gene regulatory circuit (GRC) consisting of master regulators, including transcription factors (TFs), such as ZEB, SNAIL, TWIST, and GRHL2, and microRNAs, such as miR200 and miR34. Remarkably, the core GRCs explain the existence of hybrid EMT cellular states (Lu et al., 2013). Although a generic gene regulatory network is expected for the same process in different contexts, the specific gene regulatory interactions that occur in an EMT could vary for different cell types, signaling states, and disease states (Cook and Vanderhyden, 2019). Indeed, an EMT can be induced by activating either one of the common signaling pathways, including TGFβ, EGF, TNF, Wnt, Notch, and Hedgehog signaling (Gonzalez and Medici, 2014; Steinway et al., 2014; Basu et al., 2018; Font-Clos et al., 2018). EMT has also been widely studied and observed in various types of cancer (Chung et al., 2016; Bartoschek et al., 2018; Brabletz et al., 2018), with different genetically modified mouse models (Zheng et al., 2015; Kersten et al., 2017; Chen et al., 2018) and a broad spectrum of cancer cell lines (Steinway et al., 2014; Chung et al., 2016; Bartoschek et al., 2018; Brabletz et al., 2018). Yet, little efforts have been made to identify the common and context-specific regulators and regulatory interactions during EMT and how these regulatory relationships contribute to the diversity of EMT. This investigation will help to further understand the regulatory mechanisms of EMT, elucidate the composition and stability of the various EMT states, and facilitate the discovery of new therapeutic drugs in different contexts.

Recent single-cell RNA sequencing (scRNA-seq) technology has enabled measurement of genome-wide gene expression at the single cell level. It is particularly relevant to this study, as single-cell data can not only reveal heterogeneity within cell populations but, when combined with time-series analysis, can provide a comprehensive view of the dynamics of EMT. For example, single-cell sequencing has been used to understand the intratumoral variation in cell localization and function, potentially unveiling biomarkers or drug targets (Patel et al., 2014; Bartoschek et al., 2018). A 2018 study observed a hybrid EMT state occurring during mouse organogenesis, identifying tissue type-specific regulatory elements in EMT such as *Prrx1* and *Lef1* (Dong et al., 2018). Another recent investigation found both broadly conserved regulatory elements of EMT and highly variable transcriptomic features using scRNA-seq on a melanoma dataset (Wouters et al., 2019).

A recently published dataset from Cook and Vanderhyden (2019) includes time-series scRNA-seq data from four different cancer cell lines (A549, DU145, MCF7, and OVCA420) undergoing EMT induced by one of three distinct signals (TGFB1, TNF, and EGF) for 7 days and subsequent MET induced by removing the corresponding signal. In this study, the different cell lines demonstrated distinct phenotypic trajectories with different TFs implicated in the process. The presence of context-dependent variations of the EMT trajectories confirms that the mechanism of EMT is not invariant with respect to the stimuli which induce it. The time-series data permits a thorough investigation of the path of cellular state transitions and GRCs driving the decision making of EMT in multiple experimental conditions.

**FIGURE 1:**
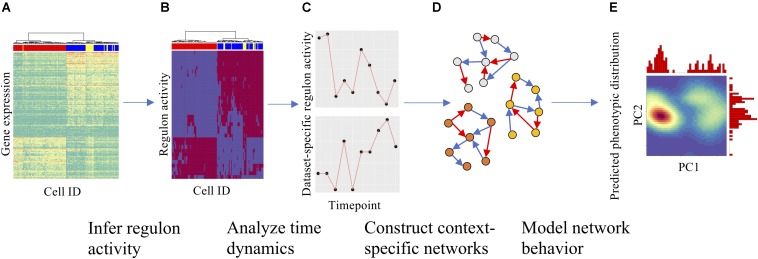
Overall strategy for analyzing scRNA-seq data and constructing context-specific gene regulatory circuits (GRCs). **(A)** Gene expression heatmap with cells in columns and gene expression levels in rows, clustered hierarchically to group cells. **(B)** Using gene expression data and SCENIC, gene regulatory module (regulon) activity for each cell could be inferred and is shown in a similar heatmap. **(C)** The time dynamics of selected regulons were then compared across datasets to identify divergent regulatory trajectories. **(D)** After the role of each regulon across datasets was characterized, numerous context-specific circuit topologies were generated (nodes represent genes, blue and red arrows represent excitatory and inhibitory regulation, respectively) using different statistical cutoffs for network modeling. **(E)** Finally, dynamics simulations were performed on each circuit to identify the optimal circuit that captures the terminal cellular states from the experimental datasets. Using simulations, one can also predict the paths of cellular state transitions upon either signal induction or removal. Density maps show PCA on simulation results with marginal histograms.

Here, we will adopt a combined bioinformatics and systems-biology modeling approach to construct context-specific core GRCs using the above-mentioned time-series scRNA-seq data from multiple cell lines and signaling treatment conditions (Figure 1). Many previous computational studies have been conducted to build EMT GRCs from literature support (Steinway et al., 2014; Hong et al., 2015; Burger et al., 2017; Huang et al., 2017; Bocci et al., 2018; Kohar and Lu, 2018; Ramirez et al., 2019). However, this approach is not optimized for the goal of this project, as there might not be sufficient literature data for a specific experimental condition. To address this issue, we systematically constructed a large number of networks for each experimental condition by starting from a collection of common TFs and integrating context-specific regulatory links derived from the gene expression data and *cis*-regulatory motif analysis. Using a wide range of network construction parameters, we evaluated the performance of each network in comparison to experimental data and generated GRCs that are highly representative of specific experimental conditions. To achieve this, a mathematical modeling method named *Ra*ndom *Ci*rcuit *Pe*rturbation (RACIPE) (Huang et al., 2017; Kohar and Lu, 2018) was applied on each network model to simulate the gene expression profiles and quantitatively compare with experimentally observed gene expression profiles. RACIPE is a parameter agnostic ordinary differential equation-based method to simulate gene regulatory networks. RACIPE takes a network topology specifying the regulator, target, and interaction type (excitatory/inhibitory) as the input and generates a large ensemble of models, where kinetic parameters of the models are randomized within a range of possible values. By simulating each of these models, RACIPE generates stable steady-state gene expression profiles from which we identify the generic features of a network and predict possible phenotypic states (see section “Materials and Methods”). From this approach, we aim to identify key regulators of EMT across all conditions as well as specific actors in each cancer type.

## Results

### Characterizing the Heterogeneity of Transcription Factor Dynamics

In this study, we focused on building GRCs using the single cell RNA sequencing (scRNA-seq) data collected from Cook and Vanderhyden (2019) for four cancer cell lines (A549, DU145, MCF7, and OVCA420) treated with EGF, TGFB1, and TNF. The data were collected at eight timepoints at 0 day, 8 h, 1 day, 3 days, and 7 days of exposure to the treatment and 8 h, 1 day, and 3 days post-signal termination at 7 days.

To evaluate the differences in the initial EMT and the backward transition occurring after the signals were removed, we separated each condition into two datasets, where the first dataset contains timepoints 0, 8 h, 1 day, 3 days, and 7 days during the signal induction, and the second dataset contains timepoints 7 days during the signal induction and 8 h, 1 day, and 3 days after the signal removal. The day 7 data were used twice here to recapitulate the dynamics in both directions. Thus, there are a collection of 24 experimental datasets in total (three treatments, four cell lines, and two directions). For each dataset, we applied SCENIC (Aibar et al., 2017) to infer the regulons or enriched transcription factors (TFs) and their corresponding TF activity for every cell. Differential analysis was then applied using Seurat (Butler et al., 2018; Stuart et al., 2019) to the activity profiles for cells at different time points. To capture the changes over time, we performed comprehensive differential activity analysis (see section “Materials and Methods”) for the forward and backward directions to obtain a list of highly variable regulons for each of the 12 conditions. Interestingly, the response to signal induction and retrieval was quite heterogeneous and only 20–30% of the differentially active TFs in the forward direction were differentially active in the backward direction as well.

Moreover, canonical EMT marker genes like SNAIL, SLUG, ZEB, and TWIST were not consistently identified as regulons in the experimental data. This finding agrees with Cook and Vanderhyden’s (2019) analysis of the datasets and suggests that complete EMT may not be taking place in the data, but with the initial response to the inductive signal leading toward partial EMT states.

### EMT Across Signaling Conditions Is Similar Within Cell Lines

**FIGURE 2:**
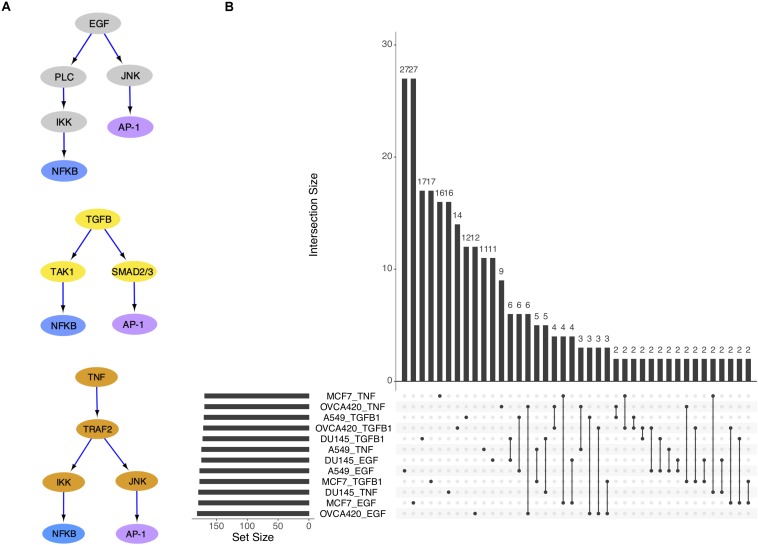
The three signal pathways have convergent gene targets. **(A)** Simplified models of signal transduction pathways for EGF, TGFB1, and TNF based on published results in the literature. **(B)** Upset plot showing overlap of differentially activated regulons across cell lines and signal treatments.

With the eventual goal of building context-specific GRCs, we first investigated which type of context was more relevant between the cell lines and the treatments. Our expectation was that the same cell line triggered by different signals may exhibit a varying response in relation to signal strength, but the nature of the transition will remain consistent across the signaling conditions. Extensive evidence exists to suggest that the three signal molecules examined in the dataset, i.e., TGFB1, EGF, and TNF, act on many of the same targets in EMT, most notably NF-κB, which comprises NFKB1 and RELB genes (Pires et al., 2017), and the AP-1 complex, which comprises FOS and JUN genes (Sun and Carpenter, 1998; Chen and Davis, 2003; Wu and Zhou, 2010; Romagnoli et al., 2012; Freudlsperger et al., 2013; Vervoort et al., 2018) (Figure 2A). On the other hand, the same signal applied to different cell lines may elicit different effects because of the unique genetic profile and mutations present in each cell line.

The overlap of differentially active TFs (DATFs) across the 12 conditions was then plotted (Figure 2B). Though a large number of DATFs are unique to each condition, more DATFs were shared across treatments of a single cell line than across cell lines for the same signal, supporting our initial hypothesis that different cell lines will have more context-specific regulatory activity than different signal treatments. In the original study, the authors also reported larger overlap among the highly variable genes for cell lines compared to signaling (Cook and Vanderhyden, 2019).

**FIGURE 3:**
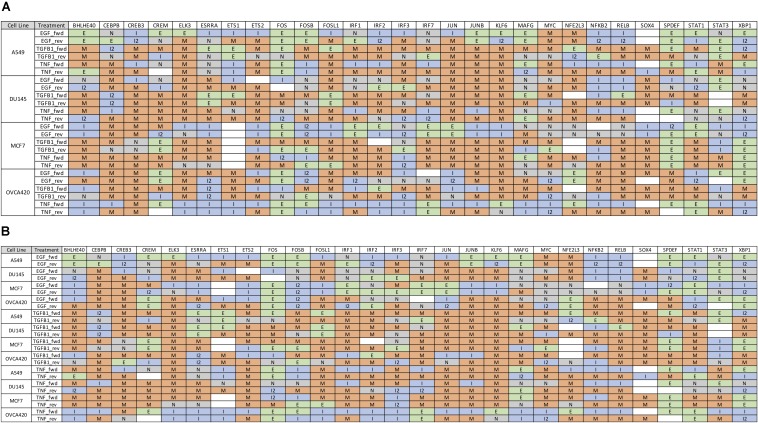
Gene expression time dynamics of common TFs for forward and backward transitions. **(A)** Table of the most common 28 TFs with state classifications sorted by cell line. While many genes play similar roles across all datasets, some, such as SPDEF and IRF3, show cell-line dependent behaviors. **(B)** 28 most common TFs with state classifications sorted by signal. As in **(A)**, some genes such as STAT1 and NFKB appear to play different roles in EMT according to the inducing signal.

**FIGURE 4:**
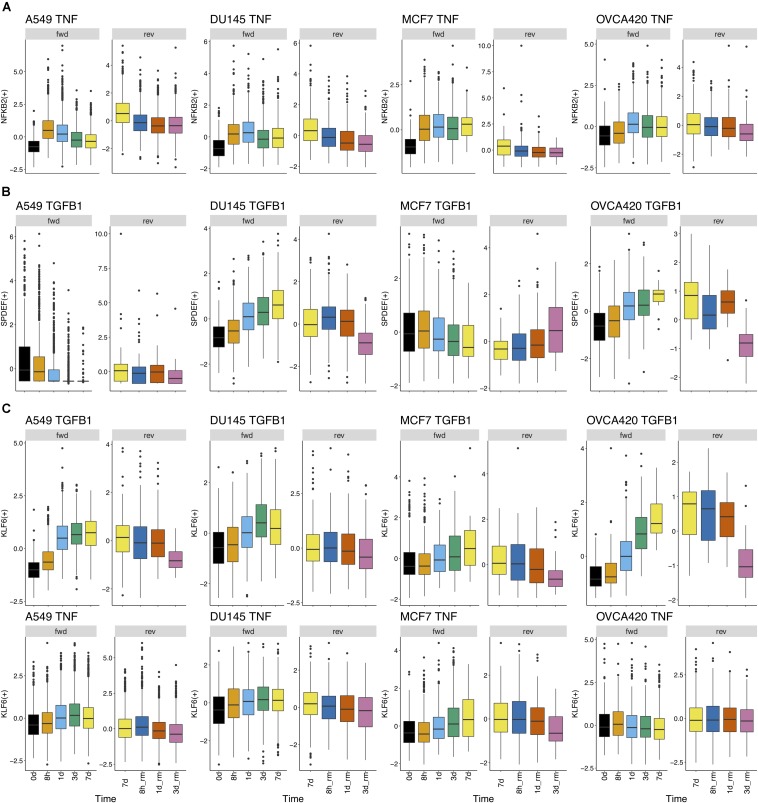
Regulon activity profiles over time. **(A)** TF activity profile for NFKB2 over time in all cell lines treated with TNF. NFKB2 generally has similar gene activity dynamics across all cell lines treated with TNF. During signal induction, NFKB2 activity initially goes up and goes down at later time points; during signal removal, it gradually decreases. **(B)** TF activity profile for SPDEF over time in all cell lines treated with TGFB1. TGFB1 dynamics differ widely across cell lines; it acts like an M gene in DU145 and OVCA420, acts as an E gene in MCF7, and shows generally low activity in A549. **(C)** TF activity profile for KLF6 over time in all cell lines treated with TGFB1 or TNF. Across both cell lines and signal treatments, KLF6 activity follows a similar pattern throughout the transition: activity largely increases during the forward transition and decreases after signal removal.

Further, we identified 28 common DATFs (Supplementary Figure S4), which frequently occur in differential analysis across timepoints (see section “Materials and Methods”). Among the most frequently identified DATFs are AP-1 genes, such as JUN and FOSL1, and NFκB genes, such as NFKB2 and RELB, consistent with the literature analysis mentioned above. Next, we annotated the TFs as E (i.e., an epithelial gene) or M (i.e., a mesenchymal gene) depending on whether the expression levels trended upward or downward over the course of the experiment. TFs whose activity increased and subsequently decreased during the transition were denoted intermediate (I) and those whose activity decreased and then increased were denoted I2 (Figures 3A,B). Across the different experimental datasets, the roles of these TFs in EMT was observed changing depending on the context (Supplementary Figure S1 for the activity time dynamics of every TF). Some TFs showed signal-specific activity profiles, such as NFKB2, which frequently served as an I gene in TNF-treated cases and an M gene in most other cases (Figure 4A). Others, such as SPDEF, showed cell line-dependent behaviors (Figure 4B). SPDEF only acted as a consistent M gene in OVCA420 and DU145, showing more E-like activity profiles in A549 and MCF7. Other genes from the overlapping TFs were more consistent across all contexts (Figure 4C); KLF6 behaved as an M gene in nearly all cases. These universally consistent genes were also generally among the well-documented EMT-related TFs, such as JUN and MYC (Supplementary Table S1).

### Exploring Intermediate EMT States and Transition Paths

**FIGURE 5:**
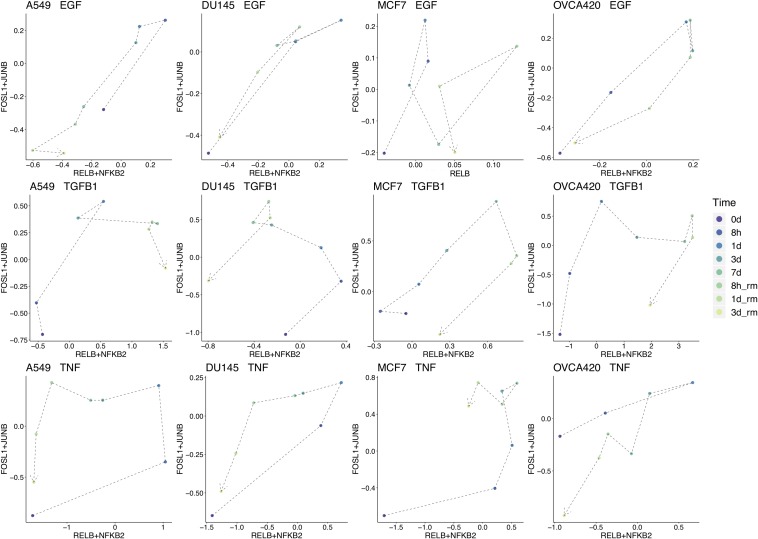
Signal chronology across experimental conditions. Combined average TF activity of FOSL1 and JUNB vs. RELB and NFKB2 for each timepoint across each cell line and signal treatment. TF activity from 7 days onward (data from the backward direction) is scaled on a linear model to match the 7 days distributions for the forward direction. Some aspects of the EMT-MET trajectory are similar across cell lines, such as in A549, DU145, and MCF7 treated with TNF, which all follow a generally counterclockwise movement. On the other hand, the transition path is also in large part determined by cell line for all signaling conditions, such as in OVCA420, where the trajectory is generally clockwise for all inducing signals.

To break down the chronological progression of EMT and the backward transition, the activity of AP-1 and NF-KB across the different timepoints were examined (Figure 5 for FOSL1 and JUNB vs. RELB and NFKB2, Supplementary Figure S2 for RELB vs. FOSL1, and Supplementary Figure S3 for RELB vs. JUNB). In multiple experimental conditions, cells exhibited an increase in activity of one signaling component before the next. During the backward transition, the order of the changes in activity is usually not consistent with the order for the forward transition, suggesting that EMT is not reversible and instead must transit through multiple distinct intermediate states depending on the direction of transition (Figure 5). The exact trajectory of the transition also varied with respect to both signaling treatment and cell line, confirming that context-specific features of EMT are present for both variables.

### Constructing Context-Specific GRCs

Next, we constructed context-specific GRCs using a combined bioinformatics and mathematical modeling protocol. Each context-specific gene network was built based on the following rules (see section “Materials and Methods” for details). First, we started with the 28 common TFs that we previously identified from comparisons across time points and identified neighboring nodes of these TFs using SCENIC regulons, i.e., genes directly upstream or downstream of the TFs. Regulatory interactions between these nodes were scored based on mutual information (MI) of TF activities, and the sign (i.e., activation or inhibition) was determined based on the sign of the TF activity Spearman’s correlations. Second, the networks for the forward and backward transitions were combined to form a unified network. Third, any TF which has only outgoing links was removed. This step was performed only once, and TFs that had only outgoing links afterward were kept in the model.

We generated a series of gene networks for each condition by varying the cutoff values of MI. From our initial exploration, we found that activating links were favored in the network construction, probably because of the nature of scRNA-seq data (Sanchez-Taltavull et al., 2019). To select different numbers of activating and inhibitory links, we varied the MI cutoffs for positive and negative interactions. These thresholds facilitated the construction of diverse networks having different number of TFs, interactions, ratio of positive and negative interactions, etc. We did an initial screening to ensure that a network contains at least 5 E or M TFs for that specific condition. However, we still investigate networks even when only positive or negative interactions are present.

**FIGURE 6:**
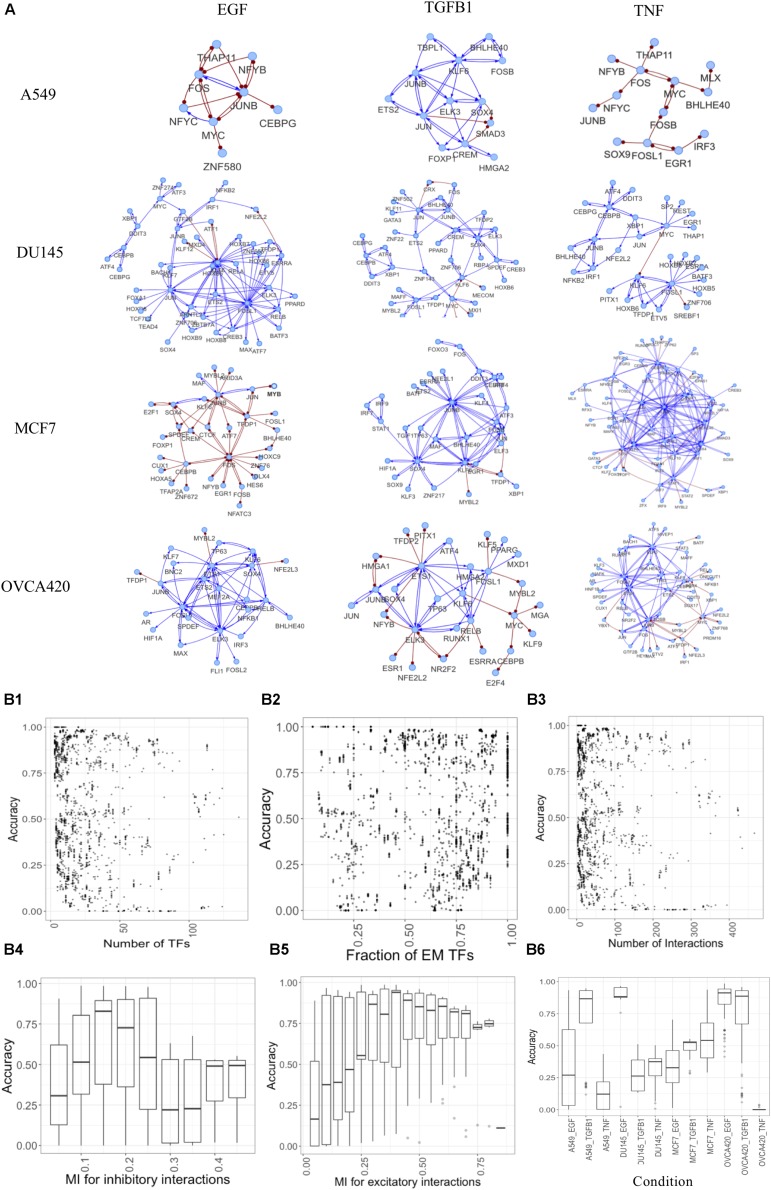
Constructing context-specific gene regulatory networks. **(A)** Representative networks yielding high accuracy scores for various conditions. Activating interactions denoted as blue arrows and inhibitory interactions as red round-tipped arrows. **(B)** Accuracy dependence on various network properties for all conditions. **(B1–B3)** Points showing accuracy (measured as fraction of models that can be classified as E or M) of models for various **(B1)** number of TFs in the network, **(B2)** fraction of TFs assigned as E or M using experimental data, **(B3)** number of interactions in the network. **(B4–B6)** Box plots showing accuracy of networks for various **(B4)** mutual information cutoffs for inhibitory interactions, **(B5)** mutual information cutoffs for excitatory interactions, **(B6)** different experimental conditions.

The generated networks were simulated using the parameter-agnostic random circuit perturbation (RACIPE) approach. We evaluated the quality of gene networks by comparing the simulated and experimental gene expression data (see section “Materials and Methods”). From this extensive analysis, we identified representative GRCs containing both E and M TFs for each condition and yielding high accuracies (Figure 6A, network topology files are listed in Supplementary Material). These networks illustrate the heterogeneity in responses for different cell lines and treatments. Simulating the large number of networks provides unique insights into network structure and resultant dynamics (Figure 6B). We found that typically moderately sized networks have better accuracy compared to large or small networks. This is reflected in the accuracy plots for various number of nodes (Figure 6B1) or interactions in the network (Figure 6B3). Expectedly, accuracy increases if the fraction of nodes that can be assigned as E or M increases (Figure 6B2), as such networks capture a larger proportion of differentially active regulons. Similarly, very low or high mutual information cutoffs for inhibitory or excitatory interactions yield lower accuracies as the network becomes sparsely connected or very dense in such cases (Figures 6B4,B5). We observed that the accuracies are also context-specific, as shown in Figure 6B6 and the performance for individual dataset shown in Supplementary Figure S5. More information on each network is given in Supplementary Table S3.

**FIGURE 7:**
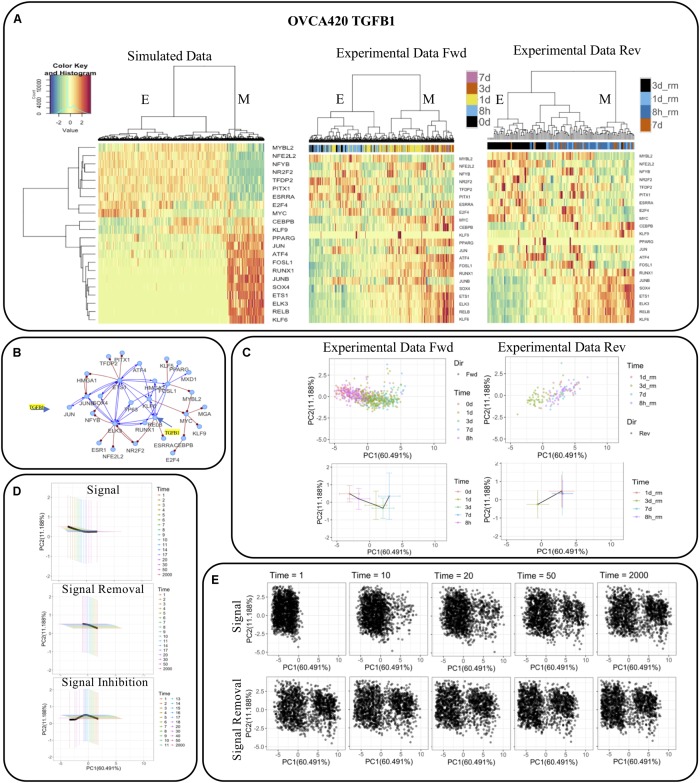
Comparison of network simulations on 2000 RACIPE models with experimental observations for OVCA420 treated with TGFB1 signaling. **(A)** Hierarchical clustering of simulated and experimental activities. **(B)** The network topology, in which TGFB1 signaling is applied to JUN and RELB. **(C)** Experimental activities (top) and their mean and standard deviation (bottom) during signal induction (left) and removal (right) projected on first two principal components of the simulated data. **(D)** Mean and standard deviation of the simulated profiles of the E-state models during signal induction (top), removal (middle), and inhibition (bottom) at multiple time points projected on the first two principal components as in **(C)**. **(E)** Simulated profiles of the E-state models during signal removal and induction at multiple time points projected on the first two principal components as in **(C)**.

Taking the GRC from the OVCA420 TGFB1 condition [identified as having the highest EMT score by Cook and Vanderhyden (2019)] as an example (Figure 7B), we directly compared the simulation results from RACIPE with the experimental data (Figure 7A). We observed that the simulations not only capture the two major E and M states, but the simulated expression profiles are also very similar to the activities of the TFs in the datasets for the forward and backward transitions (Figure 7A). We projected the experimental activities on the first two principal components of the simulated data and observed that the projected values identify the transition of cells from E to M upon signal induction and M to E during signal removal (Figure 7C). The average and standard deviation of the cells at each time point are shown in bottom panels highlighting the distinct trajectories during the forward and reverse transitions. Further, we observed that cells could not undergo a complete backward transition upon signaling removal.

To test whether we can identify similar features in our simulations, we used the E state models and applied a signal (i.e., TGFB1) to JUN and RELB and observed how the gene expressions change over time (Figure 7E). We observed the E models gradually shift toward M over time, while some models undergo a complete transition to the M state. The number of models that transit to the M state depends on the strength of the signal and noise in the simulations. The strength of the signal induction and noise were selected so that the E-state models have significantly more transitions to the M state with both signal activation and noise than those for the cases with only signal activation or noise (Supplementary Figure S7). We also observe that signal removal doesn’t result in MET in all the models that underwent EMT. When we inhibited the models by reducing the production rates of JUN and RELB below their original values, a larger fraction of models was able to transit back to the E state. The mean and standard deviation of the models at different times follow patterns quite similar to experimental activities and capture the distinct forward and backward trajectories. We found similar results when the statistics were performed to the subset of models that transit from the E state to the M state (Supplementary Figure S8). We also observed that the transition occurred at different time scales in both experiments and simulations where the cells (models) moved faster in the forward direction and slower in backward direction. The average expression of each TF at multiple time points in OVCA420 TGFB1 signal induction and removal in experiments and in simulations during signal induction, removal, and inhibition is shown in Supplementary Figure S6. Overall, these results highlight that our simulations are able to capture many aspects of experimental data.

### A Common Gene Regulatory Circuit Driving a Multi-Step EMT

**FIGURE 8:**
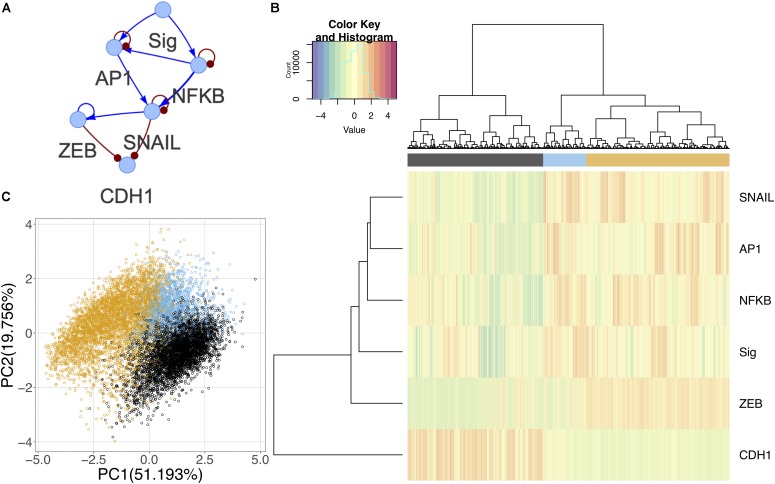
Core network simulations with 2000 RACIPE models. **(A)** Core EMT network derived from published experimental results. Activating interactions denoted as blue arrows and inhibitory interactions as red round-tipped arrows. **(B)** Heatmap of core network simulation results with ward.D2 hierarchical clustering of models and genes (number of clusters *k* = 3). **(C)** PCA of core network simulation results color coded by cluster.

Although the canonical master regulators of EMT such as SNAIL and ZEB were not prevalent in the differential activity analysis, there is evidence suggesting that they are downstream targets of the signaling pathways triggered in the experiment (Supplementary Table S2) (Chen and Davis, 2003; Li et al., 2010; Wu and Zhou, 2010; Romagnoli et al., 2012). Using information from the literature and TFs identified in the experimental data, we constructed a core GRC to model the generic effect of the signals on driving EMT (Figure 8A). Using RACIPE, we performed network simulations and identified three distinct states. One state corresponds to E cells with high expression of CDH1 and low signal strength; as the signal strength increases, models are likely to enter one of the other states: an intermediate state where signal strength is high and NF-KB and AP-1 are expressed, but ZEB remains low, and finally a full M state with high expression of M marker genes (Figure 8B). The states in this network support the hypothesis that the EMT undergone in this experiment may not be complete and may only demonstrate an initial signaling response. On a PCA plot, the intermediate state occurs between the two extreme phenotypes in one corner of the plot (Figure 8C). It is possible that other intermediate states exist when cells undergo a different type or direction of EMT, and these context-specific states are simply not captured by the common core circuit.

## Discussion

In this study, we analyzed a recent collection of time-series single cell RNA sequencing (scRNA-seq) data sets for four different cancer cell lines and three types of treatments targeting different signaling pathways to model context-specific GRCs driving EMT. We developed a combined bioinformatics and mathematical modeling approach and explored its effectiveness in constructing GRCs that capture the essential temporal dynamics derived from the scRNA-seq data. We used bioinformatics analysis to construct networks of differentially active transcription factors using the transcription factor activities obtained through co-expression and *cis*-regulatory motif analysis and used the ODE-based mathematical modeling method RACIPE to simulate the gene expression of a large number of constructed networks. The consistency of experimental activities and simulated expressions of the transcription factors was used to evaluate the networks and identify optimal networks. Our study sheds light on the regulatory mechanisms of EMT that are common and context-specific and how the identified transcriptional regulators contribute to driving or reversing EMT.

In particular, we explored the options to construct GRCs directly from *cis*-regulatory motif analysis using gene expression data and subsequent *in silico* validation by comparing circuit simulations with experimental data. From our analysis, we found it is still challenging to build high-quality circuit models directly from bioinformatics tools, consistent with a recent benchmark test (Pratapa et al., 2020). Most existing bioinformatics methods rely on statistical tests to refine network topologies by removing spurious interactions. Instead of using simple statistic-based filtering, we applied RACIPE to evaluate whether the constructed GRCs can capture the gene expression states from the data. Using RACIPE, we found a gene network typically cannot recapitulate experimentally observed cellular states when the network is either too small or too large. The optimal GRCs were mostly derived from gene networks of medium size. In addition, higher accuracy was usually found in GRCs constructed using different cutoff values for excitatory and inhibitory interactions, likely because SCENIC produced an unbalanced amount of interactions by type. We expect that an iterative procedure between network building and modeling can further improve the quality of GRC modeling.

Both the bioinformatic analysis on the data sets from multiple conditions and the literature analysis indicate that the TGFB1, EGR, and TNF signaling pathways all converge to two transcription factor (TF) complexes AP-1 and NFκB. The activation of these two complexes induces a cellular state transition to an intermediate EMT state, an event presumably occurring prior to the induction of typical EMT master regulators, such as SNAIL, TWIST, and ZEB. Our findings are consistent with the picture of multi-step state transitions during EMT (Zhang and Weinberg, 2018). One way to further test the model is to inhibit AP-1 and NFκB and evaluate how the perturbation affects EMT and MET. Moreover, from both of our bioinformatics and mathematical modeling analyses, we found that the trajectories of the forward and backward transitions do not overlap but go along two different paths, a typical hysteresis phenomenon of a non-linear dynamical system (Kramer and Fussenegger, 2005). The distinct paths of EMT and MET can be clearly illustrated by the temporal activity dynamics of AP-1 and NFκB. Such an irreversible behavior has been also observed in lung cancer in a recent study (Karacosta et al., 2019). Also, from mathematical modeling, we found that, after the initial signaling induction to achieve the forward transition, signaling removal does not fully reverse the process, but signaling inhibition can. The incomplete reverse process is also evident from the single cell data for most conditions in this study. Further characterizing the transitional paths will expand our knowledge on driving or reversing EMT.

Further, we found the performance of network modeling is context dependent. For instance, accuracies for some conditions like OVCA420 TNF were quite low. This can happen if the identified common TFs are not differentially activated, resulting in low number of E and M TFs. Cook and Vanderhyden (2019) indeed discussed that the A549 TGFB1 and OVCA420 TNF conditions had low EMT scores. Another limitation of the current approach is that it relies on SCENIC for identifying regulons and thus utilizes regulatory interactions identified by only gene co-expression and *cis*-regulatory motif enrichment analysis. One way to improve the analysis is to incorporate regulatory interactions from the literature. The specific datasets analyzed here contain heterogeneous clusters, where cells from different time points do not fall into distinct clusters (Supplementary Figure S9), but are rather on a continuum; this can also limit the robustness of the analysis. Another potential caveat of the current approach is that transcriptomics data can only capture transcriptional regulations but fall short to discover new pathways of signaling induction and metabolic pathways. A potential solution is to integrate multi-omics data (Hawe et al., 2019) to improve network construction and modeling.

## Materials and Methods

### Single Cell RNA-Seq Data Processing

Processed single cell RNA seq data were downloaded from the download link provided by Cook and Vanderhyden (2019) Normalized log counts were used in pySCENIC v0.9.19 to calculate the activities of transcription factors. We used 7-species hg19 mc9nr cisTargetDBs for the enrichment analysis. The activities obtained from SCENIC analysis were used as counts in Seurat v3.1.1 for downstream analysis. The differential activity analysis was conducted using Seurat. We used default settings except for the log fold change criteria which we reduced to zero, as the activity fold change is quite low. To capture the changes over time, we performed seven comparisons for each of the twelve conditions – four for the forward group (1) 0 vs. 8 h; (2) 0 vs. 1 day; (3) 0 vs. 3 days; (4) 0 vs. 7 days; and three for the backward group (5) 7 days vs. 8 h; (6) 7 days vs. 1 day; (7) 7 days vs. 3 days. From this comprehensive differential analysis for 84 comparisons, we selected top hundred DATFs (sorted based on adjusted *p*-values) for the forward and backward directions to obtain a list of highly variable TFs for each of the twelve conditions. Further, we identified 28 DATFs, each of which occurs in at least 24 of the 84 pairwise comparisons (Supplementary Figure S5). The TFs were annotated as E (i.e., an epithelial gene) depending on whether the log fold change in the 0 day vs. 7 days (7 days vs. 3 d_rm) comparison is negative (positive) with an adjusted *p*-value of 0.05. Similarly, TFs with log fold change positive (negative) in the 0 day vs. 7 days (7 days vs. 3 d_rm) comparison are annotated as M (i.e., a mesenchymal gene). Scaled activity values from Seurat were used for network analysis. We scaled the backward activities whenever the forward and backward activities were needed on the same scale (for example, in gene activity plots in Figures 4, 7). As the 7 days cells were included in both forward and backward datasets, these were used to fit a linear model which was then used to scale the activities of cells from the other days where the signal is removed.

### Network Construction

All the nearest neighboring TFs of the common DATFs were identified using the SCENIC regulons. Thus, for a TF (annotated as TF1), if either the forward or backward regulon for the TF includes another TF (annotated as TF2), then TF2 was identified as a target of TF1. Spearman’s correlation between the activities of the TFs in a specific dataset was used for assigning an interaction as excitatory or inhibitory. Mutual information between the DATF activities was calculated with infotheo R package using “mm” correction (Meyer, 2014). The interactions in the network were filtered based on MI cut offs and any interaction with opposite sign in the forward and backward direction was removed from the network. Based on the maximum and minimum values of MI, we varied the positive MI cutoffs from 0.05 to 1 and the negative MI cutoffs from 0.05 to 0.5 incrementing by 0.05 at each step. If a TF in a network had only outgoing interactions with no incoming interactions, then the TF was removed from the network. This pruning was done only once – if removing a TF this way makes another TF with only outgoing interactions, then this new signaling TF is not removed.

### Network Simulation

The network construction step generated a large number of networks with different number of nodes and interactions for each condition. The resulting networks, which specify the interactions in the form of regulator, target, and interaction type, were simulated using the default settings in sRACIPEv1.3.1 (Huang et al., 2017; Kohar and Lu, 2018). Specifically, 2000 models with randomized kinetic parameters were generated for each network. The model kinetic parameters include two parameters for each gene – maximum production rate (1–100) and degradation rate (0.1–1) and three parameters for each interaction – Hill coefficient of co-operativity (1–6), fold change (1–100), and threshold. The numbers in brackets indicate the range from which the corresponding parameter was selected. The range for threshold for interactions was dynamically selected based on network topology to roughly satisfy the half functional rule (Huang et al., 2017). The initial condition for each gene in a model was selected from a log distribution over the minimum and maximum possible expression value for that gene in the model with given kinetic parameters. For more details, please refer to Huang et al. (2017). The ODEs with these kinetic parameters and initial conditions were solved using the fourth order Runge–Kutta method and the model state was recorded after 50 time units. These simulated gene expressions for all the models were log transformed and standardized for further analysis.

### Network Evaluation

Gene networks were evaluated by comparing the simulated and experimental TF activity data. For each experimental condition, the activities at 0 and 7 days during signal induction and 3 days after signal removal were used to classify the network TFs as either E or M TFs. For the forward transition, a TF was defined as an M TF if the gene has differential activity (adjusted *p*-value < 0.05) with a positive log fold change when comparing the data from 0 and 7 days, and as an E TF if the log fold change is negative. Similarly, for the backward transition, a TF was defined as an M TF if the gene has differential activity with a negative log fold change when comparing the data from 7 and 3 days after signal removal, and as an E TF if the log fold change is positive. The EMT states were not assigned to TFs if there is a conflict between E and M assignment for the forward and backward transitions. Using these E and M classification, we defined two digitized gene expression vectors as references to represent the E and M states. Here, in the E reference state, E TFs have high expressions (denoted as 1) and M TFs have low expressions (denoted as 0), vice versa. These E and M reference states were used to identify whether the 2000 simulated profiles using RACIPE can generate models in the E and M states. The simulated profiles were binarized with the binarize R package and kMeans method with two clusters (Mundus et al., 2019). The fraction of models having expression profiles close to the E and M state were calculated to evaluate how accurately the constructed network can capture the EMT states. The similarity between the binarized expression profiles and the digitized E and M expression vectors was measured by calculating the hamming distance between the common TFs. The hamming distance cutoff for matching of simulated and experimental data is selected based on number of common TFs such that the probability of a match by random chance stays below 0.05.

To model the signal induction in simulations, we selected all of the E models obtained in the previous simulations and increased the production rates of JUN and RELB in the OVCA420 TGFB1 network. The productions rates of both were multiplied by 10,000 and the network was simulated keeping the other parameters same (Figures 7D,E). Steady state solutions obtained from previous simulations were used as the initial conditions. The trajectories were sampled at multiple time points to capture the dynamics. To account for intrinsic and extrinsic noise and facilitate the transition between the states we also added some noise (0.05) during the simulations. Then, the production rates were reverted back to their original values to reflect the removal of signals. In another set of simulations, using the steady state solutions of the signal induction models as the initial condition, the production rates of RELB and JUN were decreased (multiplied by 0.0001) below their original values to allow all the E state models to transit back (Figures 7D,E).

## Data Availability Statement

All sequencing data used in this study was generated by Cook and Vanderhyden (2019) and is available at https://drive.google.com/drive/folders/1lZ38Uj2ZjmFus7XbHGTATh8f9MqXLAf_. The python and R scripts used to perform the analyses here are available at https://lusystemsbio.github.io/EMT-Cancer-scRnaSeq/EMT-Cancer-scRnaSeq.html.

## Author Contributions

ML conceived the scope and design of the study. DR and VK conducted experimental data analysis and figure generation. VK generated network models and performed simulations. DR, VK, and ML contributed to drafting and revising the manuscript.

## Conflict of Interest

The authors declare that the research was conducted in the absence of any commercial or financial relationships that could be construed as a potential conflict of interest.

## References

[B1] Aibar S. González-Blas C. B. Moerman T. Huynh-Thu V. A. Imrichova H. Hulselmans G. (2017). SCENIC: single-cell regulatory network inference and clustering. *Nat. Methods* 14 1083–1086. 10.1038/nmeth.4463 28991892 PMC5937676

[B2] Bartoschek M. Oskolkov N. Bocci M. Lövrot J. Larsson C. Sommarin M. (2018). Spatially and functionally distinct subclasses of breast cancer-associated fibroblasts revealed by single cell RNA sequencing. *Nat. Commun.* 9:5150. 10.1038/s41467-018-07582-3 PMC6279758 30514914

[B3] Basu S. Cheriyamundath S. Ben-Ze’ev A. (2018). Cell–cell adhesion: linking Wnt/β-catenin signaling with partial EMT and stemness traits in tumorigenesis. *F1000Res* 7:1488. 10.12688/f1000research.15782.1 30271576 PMC6144947

[B4] Bocci F. Jolly M. K. George J. T. Levine H. Onuchic J. N. (2018). A mechanism-based computational model to capture the interconnections among epithelial-mesenchymal transition, cancer stem cells and Notch-Jagged signaling. *Oncotarget* 9 29906–29920. 30042822 10.18632/oncotarget.25692 PMC6057462

[B5] Brabletz T. Kalluri R. Nieto M. A. Weinberg R. A. (2018). EMT in cancer. *Nat. Rev. Cancer* 18 128–134. 29326430 10.1038/nrc.2017.118

[B6] Burger G. A. Danen E. H. J. Beltman J. B. (2017). Deciphering epithelial–mesenchymal transition regulatory networks in cancer through computational approaches. *Front. Oncol.* 7:162. 10.3389/fonc.2017.00162 28824874 PMC5540937

[B7] Butler A. Hoffman P. Smibert P. Papalexi E. Satija R. (2018). Integrating single-cell transcriptomic data across different conditions, technologies, and species. *Nat. Biotechnol.* 36 411–420. 10.1038/nbt.4096 29608179 PMC6700744

[B8] Chen D. Davis J. S. (2003). Epidermal growth factor induces c-fos and c-jun mRNA via Raf-1/MEK1/ERK-dependent and -independent pathways in bovine luteal cells. *Mol. Cel. Endocrinol.* 200 141–154. 10.1016/s0303-7207(02)00379-9 12644307

[B9] Chen Y. LeBleu V. S. Carstens J. L. Sugimoto H. Zheng X. Malasi S. (2018). Dual reporter genetic mouse models of pancreatic cancer identify an epithelial-to-mesenchymal transition-independent metastasis program. *EMBO Mol. Med.* 10:e9085. 10.15252/emmm.201809085 PMC6180301 30120146

[B10] Chung V. Y. Tan T. Z. Tan M. Wong M. K. Kuay K. T. Yang Z. (2016). GRHL2-miR-200-ZEB1 maintains the epithelial status of ovarian cancer through transcriptional regulation and histone modification. *Sci. Rep.* 6:19943. 10.1038/srep19943 PMC4757891 26887977

[B11] Cook D. P. Vanderhyden B. C. (2019). Comparing transcriptional dynamics of the epithelial-mesenchymal transition. *Cancer Biol. biorxiv* [Preprint]. 10.1101/732412

[B12] Ding S. Zhang W. Xu Z. Xing C. Xie H. Guo H. (2013). Induction of an EMT-like transformation and MET in vitro. *J. Transl. Med.* 11:164. 10.1186/1479-5876-11-164 23829659 PMC3716679

[B13] Dong J. Hu Y. Fan X. Wu X. Mao Y. Hu B. (2018). Single-cell RNA-seq analysis unveils a prevalent epithelial/mesenchymal hybrid state during mouse organogenesis. *Genome Biol.* 19:31 10.1186/s13059-018-1416-2 PMC5853091 29540203

[B14] Font-Clos F. Zapperi S. La Porta C. A. M. (2018). Topography of epithelial–mesenchymal plasticity. *Proc,. Natl. Acad. Sci. U.S.A.* 115 5902–5907. 10.1073/pnas.1722609115 29784817 PMC6003369

[B15] Freudlsperger C. Bian Y. Contag Wise S. Burnett J. Coupar J. Yang X. (2013). TGF-β and NF-κB signal pathway cross-talk is mediated through TAK1 and SMAD7 in a subset of head and neck cancers. *Oncogene* 32 1549–1559. 10.1038/onc.2012.171 22641218 PMC3434281

[B16] Gonzalez D. M. Medici D. (2014). Signaling mechanisms of the epithelial-mesenchymal transition. *Sci. Signal.* 7:re8. 10.1126/scisignal.2005189 25249658 PMC4372086

[B17] Hawe J. S. Theis F. J. Heinig M. (2019). Inferring interaction networks from multi-omics data. *Front. Genet.* 10:535 10.3389/fgene.2019.00535 PMC6582773 31249591

[B18] Hong T. Watanabe K. Ta C. H. Villarreal-Ponce A. Nie Q. Dai X. (2015). An Ovol2-Zeb1 mutual inhibitory circuit governs bidirectional and multi-step transition between epithelial and mesenchymal states. *PLoS Comput. Biol.* 11:e1004569. 10.1371/journal.pcbi.1004569 26554584 PMC4640575

[B19] Huang B. Lu M. Jia D. Ben-Jacob E. Levine H. Onuchic J. N. (2017). Interrogating the topological robustness of gene regulatory circuits by randomization. *PLoS Comput. Biol.* 13:e1005456. 10.1371/journal.pcbi.1005456 28362798 PMC5391964

[B20] Jia D. Li X. Bocci F. Tripathi S. Deng Y. Jolly M. K. (2019). Quantifying cancer epithelial-mesenchymal plasticity and its association with stemness and immune response. *JCM* 8:725. 10.3390/jcm8050725 31121840 PMC6572429

[B21] Jolly M. K. (2015). Implications of the hybrid epithelial/mesenchymal phenotype in metastasis. *Front. Oncol.* 5:155. 10.3389/fonc.2015.00155 26258068 PMC4507461

[B22] Karacosta L. G. Anchang B. Ignatiadis N. Kimmey S. C. Benson J. A. Shrager J. B. (2019). Mapping lung cancer epithelial-mesenchymal transition states and trajectories with single-cell resolution. *Nat. Commun.* 10:5587. 10.1038/s41467-019-13441-6 PMC6898514 31811131

[B23] Kersten K. Visser K. E. Miltenburg M. H. Jonkers J. (2017). Genetically engineered mouse models in oncology research and cancer medicine. *EMBO Mol. Med.* 9 137–153. 10.15252/emmm.201606857 28028012 PMC5286388

[B24] Kohar V. Lu M. (2018). Role of noise and parametric variation in the dynamics of gene regulatory circuits. *Syst. Biol. Appl.* 4:40. 10.1038/s41540-018-0076-x PMC6218471 30416751

[B25] Kramer B. P. Fussenegger M. (2005). Hysteresis in a synthetic mammalian gene network. *Proc. Natl. Acad. Sci. U.S.A.* 102 9517–9522. 10.1073/pnas.0500345102 15972812 PMC1172236

[B26] Li Y. Liu Y. Xu Y. Voorhees J. J. Fisher G. J. (2010). UV irradiation induces Snail expression by AP-1 dependent mechanism in human skin keratinocytes. *J. Dermatol. Sci.* 60 105–113. 10.1016/j.jdermsci.2010.08.003 20851575

[B27] Lu M. Jolly M. K. Levine H. Onuchic J. N. Ben-Jacob E. (2013). MicroRNA-based regulation of epithelial-hybrid-mesenchymal fate determination. *Proc. Natl. Acad. Sci. U.S.A.* 110 18144–18149. 10.1073/pnas.1318192110 24154725 PMC3831488

[B28] Meyer P. E. (2014). *infotheo: Information-Theoretic Measures.*

[B29] Mundus S. Müssel C. Schmid F. Lausser L. Blätte T. J. Hopfensitz M. (2019). Binarize: Binarization of One-Dimensional Data. *R package version 1.3.* Available online at: https://CRAN.R-project.org/package=Binarize

[B30] Nieto M. A. Huang R. Y.-J. Jackson R. A. Thiery J. P. (2016). EMT: 2016. *Cell* 166 21–45. 27368099 10.1016/j.cell.2016.06.028

[B31] Nistico P. Bissell M. J. Radisky D. C. (2012). Epithelial-mesenchymal transition: general principles and pathological relevance with special emphasis on the role of matrix metalloproteinases. *Cold Spring Harbor Perspect. Biol.* 4:a011908. 10.1101/cshperspect.a011908 22300978 PMC3281569

[B32] Patel A. P. Tirosh I. Trombetta J. J. Shalek A. K. Gillespie S. M. Wakimoto H. (2014). Single-cell RNA-seq highlights intratumoral heterogeneity in primary glioblastoma. *Science* 344 1396–1401. 10.1126/science.1254257 24925914 PMC4123637

[B33] Pires B. R. B. Mencalha A. L. Ferreira G. M. de Souza W. F. Morgado-Díaz J. A. Maia A. M. (2017). NF-kappaB is involved in the regulation of EMT genes in breast cancer cells. *PLoS One* 12:e0169622. 10.1371/journal.pone.0169622 28107418 PMC5249109

[B34] Pratapa A. Jalihal A. P. Law J. N. Bharadwaj A. Murali T. M. (2020). Benchmarking algorithms for gene regulatory network inference from single-cell transcriptomic data. *Nat. Methods.* 17 147–154. 10.1038/s41592-019-0690-6 31907445 PMC7098173

[B35] Ramirez D. Kohar V. Katebi A. Lu M. (2019). Modeling a gene regulatory network of EMT hybrid states for mouse embryonic skin cells. *bioRxiv* [Preprint]. 10.1101/799908.

[B36] Romagnoli M. Belguise K. Yu Z. Wang X. Landesman-Bollag E. Seldin D. C. (2012). Epithelial-to-mesenchymal transition induced by TGF-β1 Is mediated by blimp-1–dependent repression of BMP-5. *Cancer Res.* 72 6268–6278. 10.1158/0008-5472.can-12-2270 23054396 PMC3513653

[B37] Sanchez-Taltavull D. Perkins T. J. Dommann N. Melin N. Keogh A. Candinas D. (2019). Bayesian Correlation is a robust similarity measure for single cell RNA-seq data. *Bioinformatics* 2:lqaa002. 10.1093/nargab/lqaa002 PMC7671344 33575552

[B38] Steinway S. N. Zanudo J. G. T. Ding W. Rountree C. B. Feith D. J. Loughran T. P. (2014). Network modeling of TGF signaling in hepatocellular carcinoma epithelial-to-mesenchymal transition reveals joint sonic hedgehog and wnt pathway activation. *Cancer Res.* 74 5963–5977. 10.1158/0008-5472.can-14-0225 25189528 PMC4851164

[B39] Stuart T. Butler A. Hoffman P. Hafemeister C. Papalexi E. Mauck W. M. (2019). Comprehensive integration of single-cell data. *Cell* 177 1888.e21–1902.e21. 31178118 10.1016/j.cell.2019.05.031 PMC6687398

[B40] Sun L. Carpenter G. (1998). Epidermal growth factor activation of NF-κB is mediated through IκBα degradation and intracellular free calcium. *Oncogene* 16 2095–2102. 10.1038/sj.onc.1201731 9572490

[B41] Thiery J. P. Acloque H. Huang R. Y. J. Nieto M. A. (2009). Epithelial-mesenchymal transitions in development and disease. *Cell* 139 871–890. 10.1016/j.cell.2009.11.007 19945376

[B42] Vervoort S. J. Lourenço A. R. Tufegdzic Vidakovic A. Mocholi E. Sandoval J. L. Rueda O. M. (2018). SOX4 can redirect TGF-β-mediated SMAD3-transcriptional output in a context-dependent manner to promote tumorigenesis. *Nucleic Acids Res.* 46 9578–9590. 10.1093/nar/gky755 30137431 PMC6182182

[B43] Wouters J. Kalender-Atak Z. Minnoye L. Spanier K. I. De Waegeneer M. González-Blas C. B. (2019). Single-cell gene regulatory network analysis reveals new melanoma cell states and transition trajectories during phenotype switching. *Genomics. bioRxiv* [Preprint].

[B44] Wu Y. Zhou B. P. (2010). TNF-α/NF-κB/Snail pathway in cancer cell migration and invasion. *Br. J. Cancer* 102 639–644. 10.1038/sj.bjc.6605530 20087353 PMC2837572

[B45] Zhang Y. Weinberg R. A. (2018). Epithelial-to-mesenchymal transition in cancer: complexity and opportunities. *Front. Med.* 12 361–373. 10.1007/s11684-018-0656-6 30043221 PMC6186394

[B46] Zheng X. Carstens J. L. Kim J. Scheible M. Kaye J. Sugimoto H. (2015). Epithelial-to-mesenchymal transition is dispensable for metastasis but induces chemoresistance in pancreatic cancer. *Nature* 527 525–530. 10.1038/nature16064 26560028 PMC4849281

