# Supplementary material for: Toward Modeling Context-Specific EMT Regulatory Networks Using Temporal Single Cell RNA-Seq Data
Source: Front Mol Biosci. 2020 Apr 23;7:54. doi: 10.3389/fmolb.2020.00054 (PMC7190801; doi:10.3389/fmolb.2020.00054)
Supplement: Supplementary file 1 [file Presentation_1.pdf]

Towards modeling context-specific EMT regulatory networks using temporal single cell RNA-Seq data

Daniel Ramirez[1*], Vivek Kohar[2*], Mingyang Lu[2]

1 College of Health Solutions, Arizona State University, Tempe AZ

2 The Jackson Laboratory for Mammalian Genetics, Bar Harbor ME

* equal contributions

Correspondence:
Mingyang Lu
Mingyang.Lu@jax.org

Supporting Information

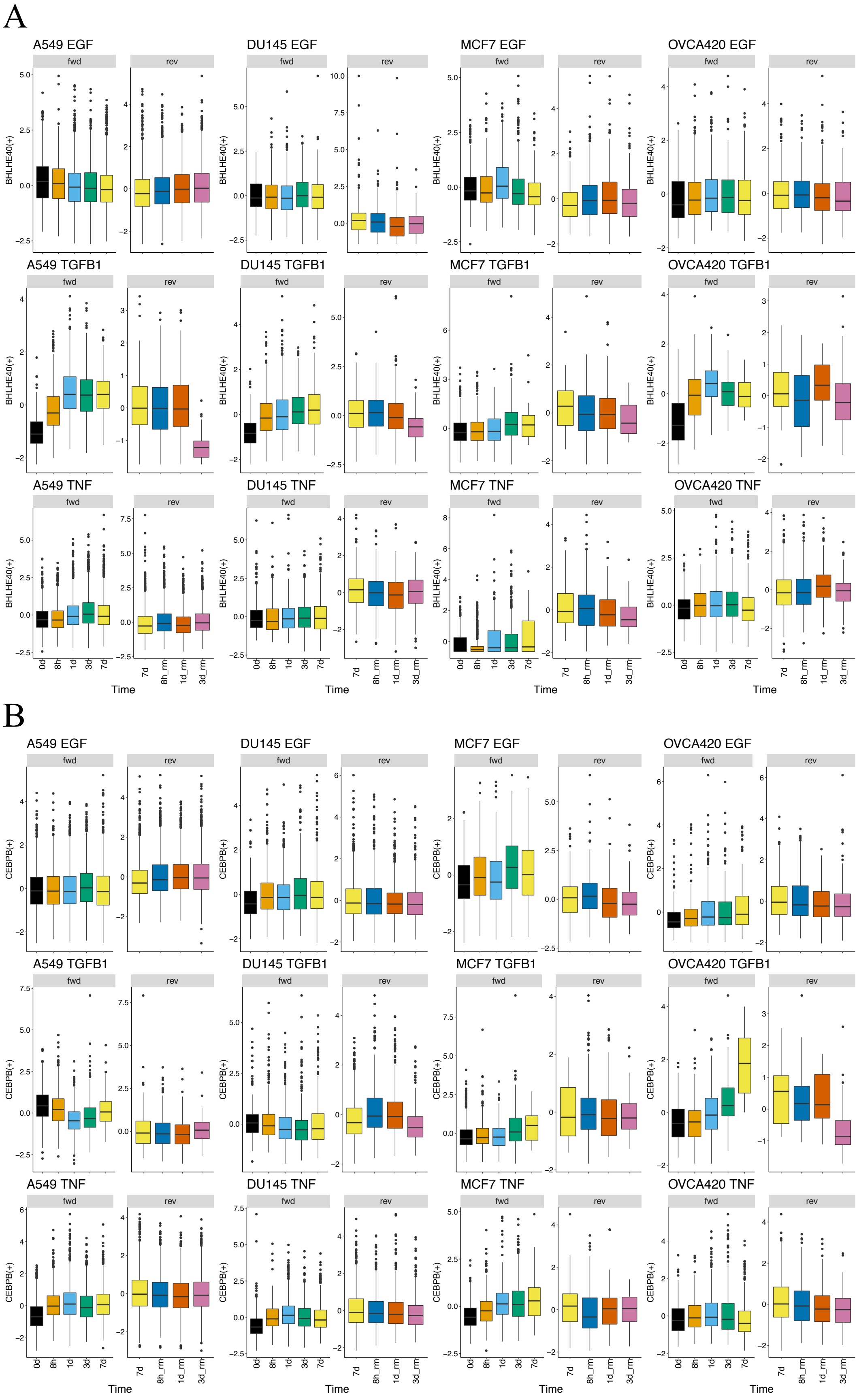

A
A549 EGF
DU145 EGF
MCF7 EGF
OVCA420 EGF
A549 TGFB1
DU145 TGFB1
MCF7 TGFB1
OVCA420 TGFB1
A549 TNF
DU145 TNF
MCF7 TNF
OVCA420 TNF
fwd
rev
BHLHE40(+)
0d
8h
1d
3d
7d
8h_rm
1d_rm
3d_rm
Time
B
A549 EGF
DU145 EGF
MCF7 EGF
OVCA420 EGF
A549 TGFB1
DU145 TGFB1
MCF7 TGFB1
OVCA420 TGFB1
A549 TNF
DU145 TNF
MCF7 TNF
OVCA420 TNF
fwd
rev
CEBPB(+)
Time

C

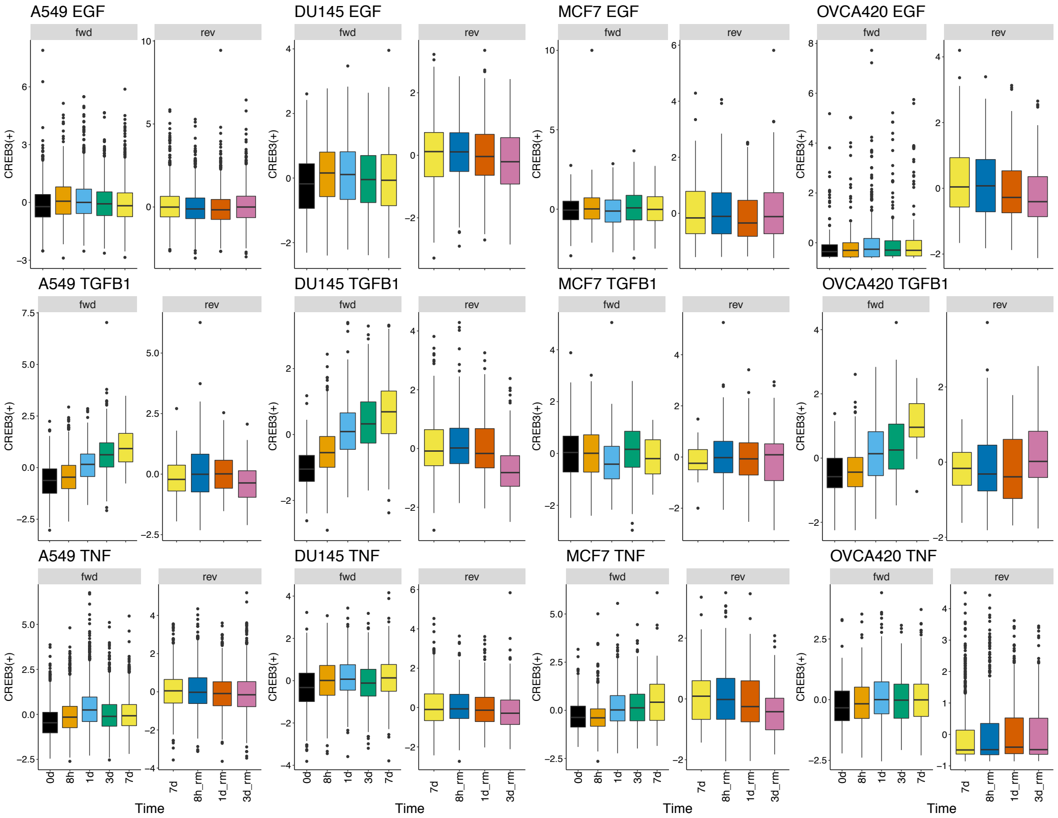
A549 EGF
DU145 EGF
MCF7 EGF
OVCA420 EGF
A549 TGFB1
DU145 TGFB1
MCF7 TGFB1
OVCA420 TGFB1
A549 TNF
DU145 TNF
MCF7 TNF
OVCA420 TNF
fwd
rev
CREB3(+)
0d
8h
1d
3d
7d
8h_rm
1d_rm
3d_rm
Time

D

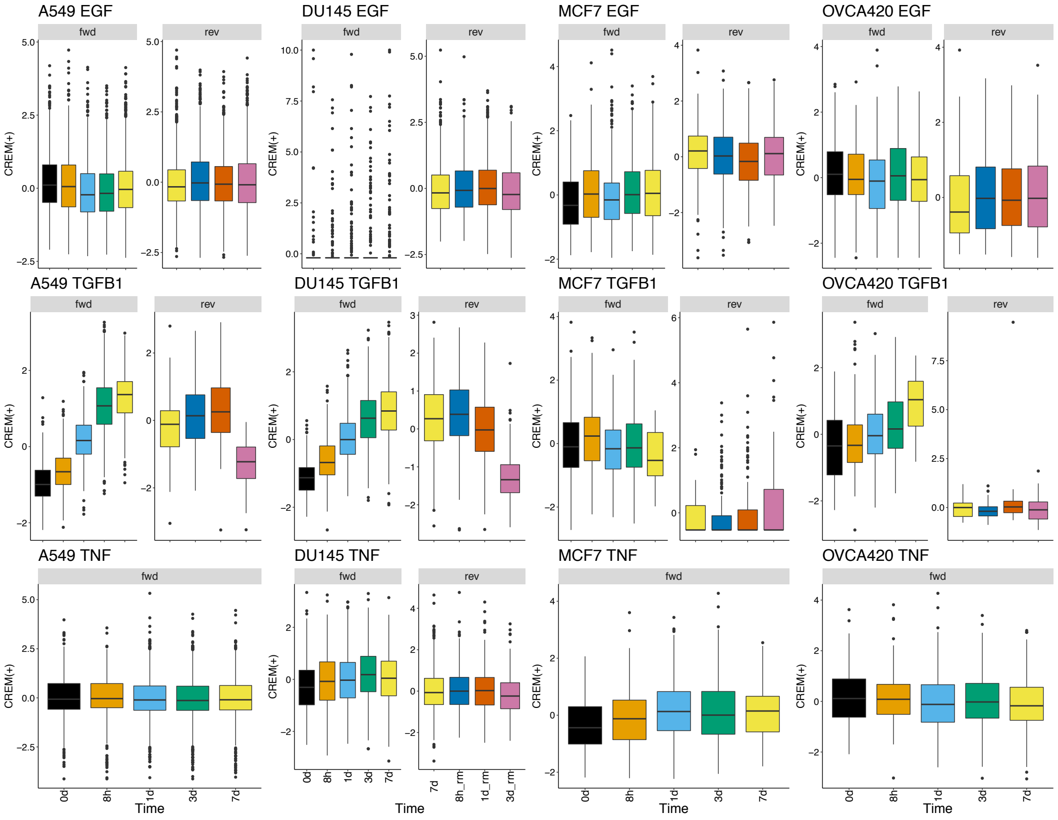
A549 EGF
DU145 EGF
MCF7 EGF
OVCA420 EGF
A549 TGFB1
DU145 TGFB1
MCF7 TGFB1
OVCA420 TGFB1
A549 TNF
DU145 TNF
MCF7 TNF
OVCA420 TNF
fwd
rev
CREM(+)
0d
8h
1d
3d
7d
8h_rm
1d_rm
3d_rm
Time

E

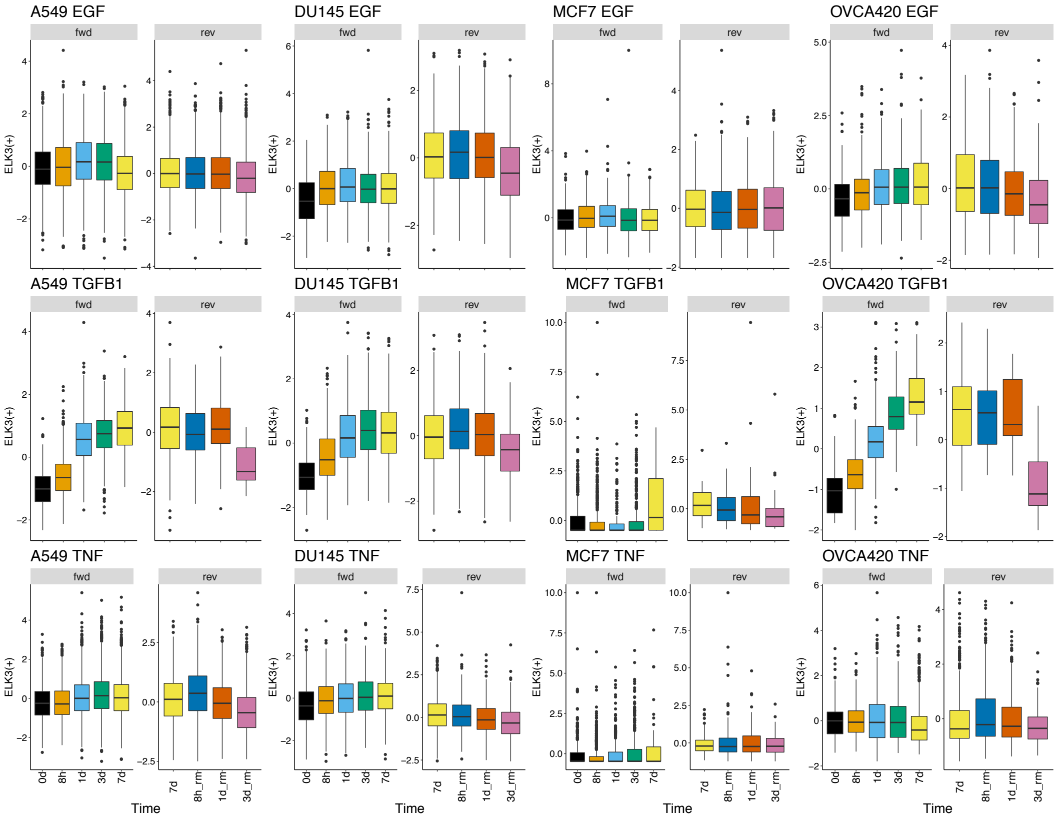
A549 EGF
DU145 EGF
MCF7 EGF
OVCA420 EGF
A549 TGFB1
DU145 TGFB1
MCF7 TGFB1
OVCA420 TGFB1
A549 TNF
DU145 TNF
MCF7 TNF
OVCA420 TNF
fwd
rev
ELK3(+)
0d
8h
1d
3d
7d
7d
8h_rm
1d_rm
3d_rm
Time

F

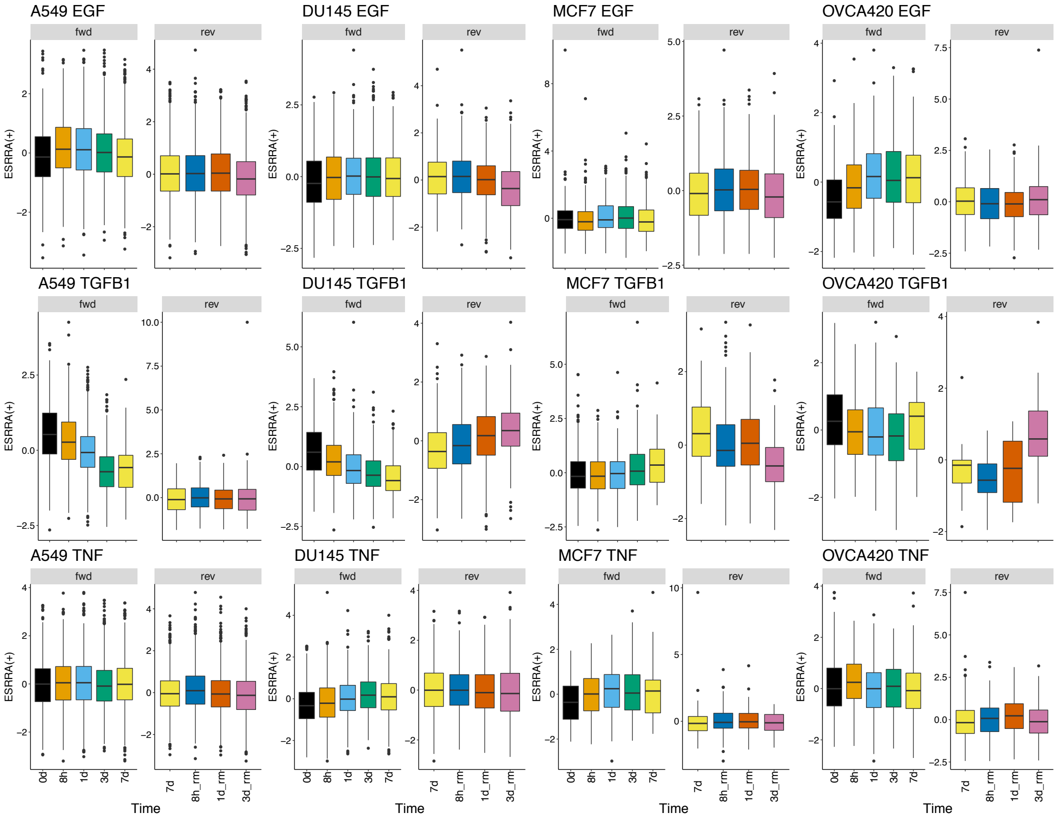
A549 EGF
DU145 EGF
MCF7 EGF
OVCA420 EGF
A549 TGFB1
DU145 TGFB1
MCF7 TGFB1
OVCA420 TGFB1
A549 TNF
DU145 TNF
MCF7 TNF
OVCA420 TNF
fwd
rev
ESRRA(+)
0d
8h
1d
3d
7d
7d
8h_rm
1d_rm
3d_rm
Time

G

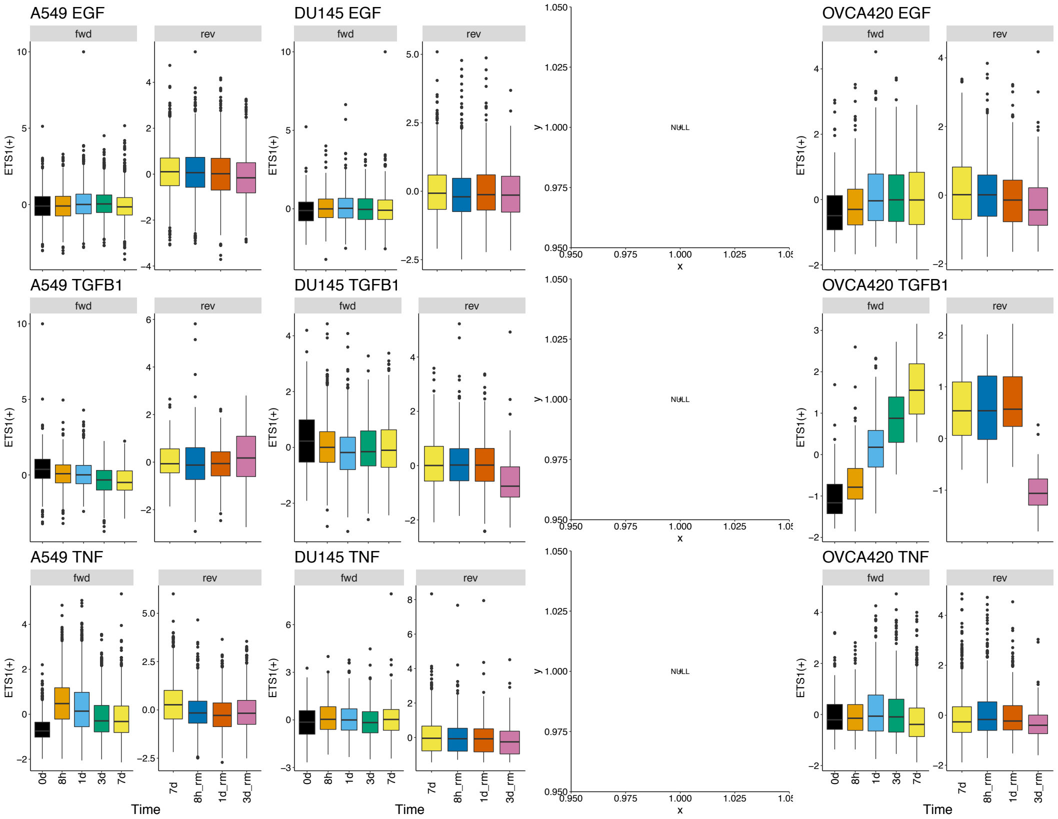

A549 EGF
DU145 EGF
OVCA420 EGF
A549 TGFB1
DU145 TGFB1
OVCA420 TGFB1
A549 TNF
DU145 TNF
OVCA420 TNF
fwd
rev
ETS1(+)
NULL
x
y
0d
8h
1d
3d
7d
8h_rm
1d_rm
3d_rm
Time

H

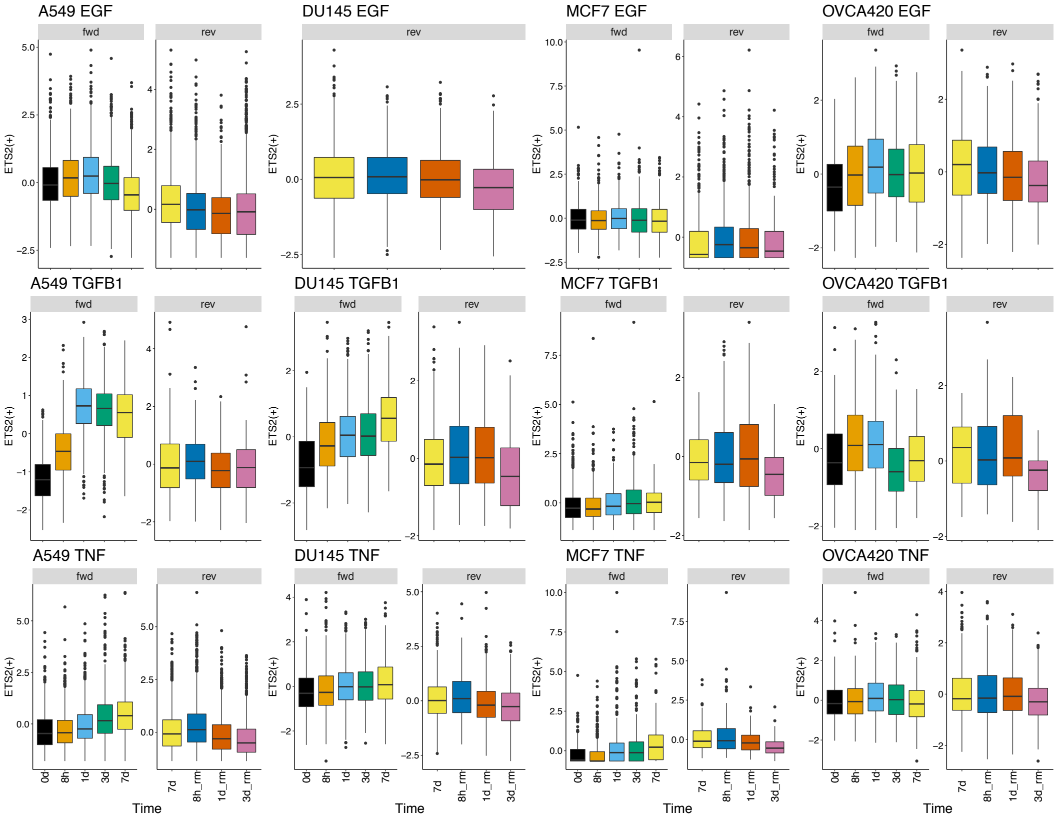

A549 EGF
DU145 EGF
MCF7 EGF
OVCA420 EGF
A549 TGFB1
DU145 TGFB1
MCF7 TGFB1
OVCA420 TGFB1
A549 TNF
DU145 TNF
MCF7 TNF
OVCA420 TNF
fwd
rev
ETS2(+)
0d
8h
1d
3d
7d
8h_rm
1d_rm
3d_rm
Time

I

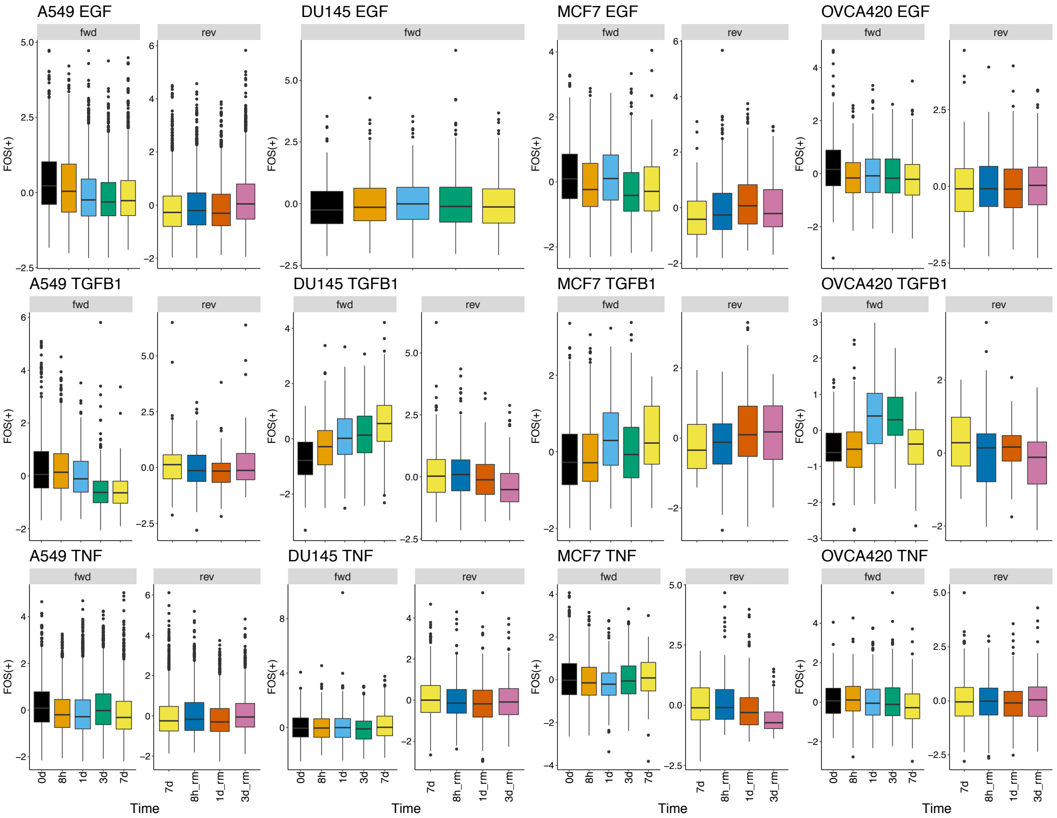
A549 EGF
DU145 EGF
MCF7 EGF
OVCA420 EGF
A549 TGFB1
DU145 TGFB1
MCF7 TGFB1
OVCA420 TGFB1
A549 TNF
DU145 TNF
MCF7 TNF
OVCA420 TNF
fwd
rev
FOS(+)
0d
8h
1d
3d
7d
8h_rm
1d_rm
3d_rm
Time

J

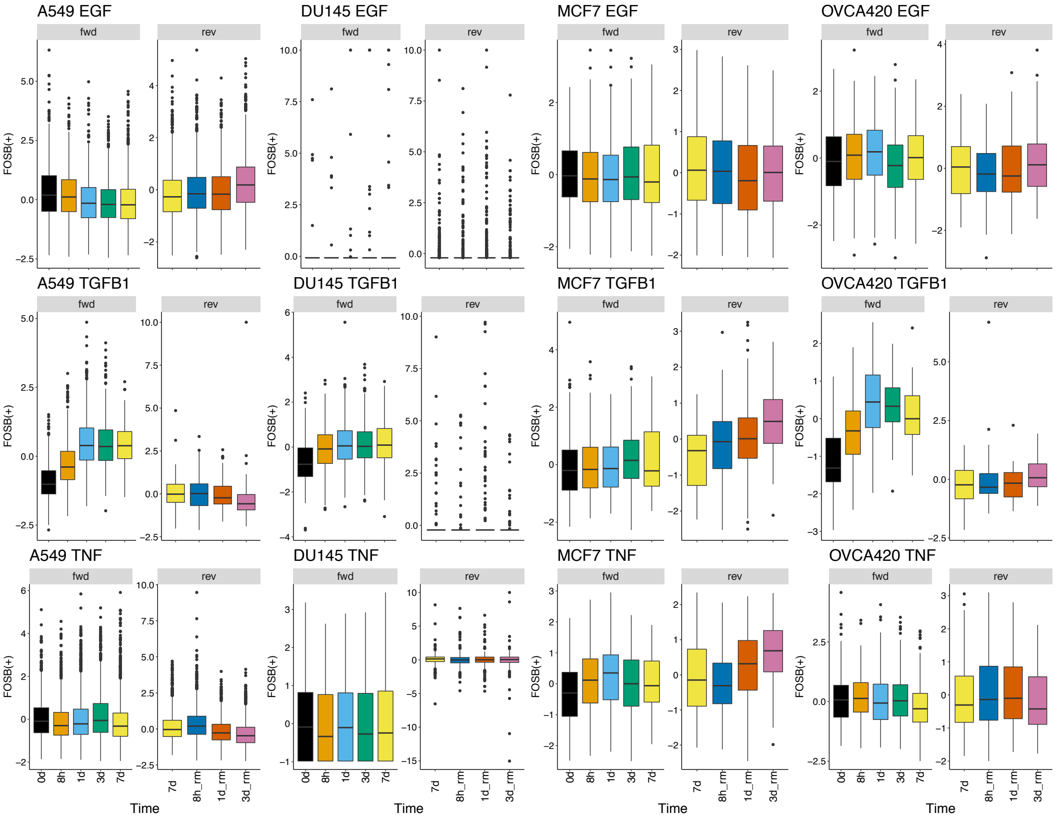
A549 EGF
DU145 EGF
MCF7 EGF
OVCA420 EGF
A549 TGFB1
DU145 TGFB1
MCF7 TGFB1
OVCA420 TGFB1
A549 TNF
DU145 TNF
MCF7 TNF
OVCA420 TNF
fwd
rev
FOSB(+)
0d
8h
1d
3d
7d
8h_rm
1d_rm
3d_rm
Time

K

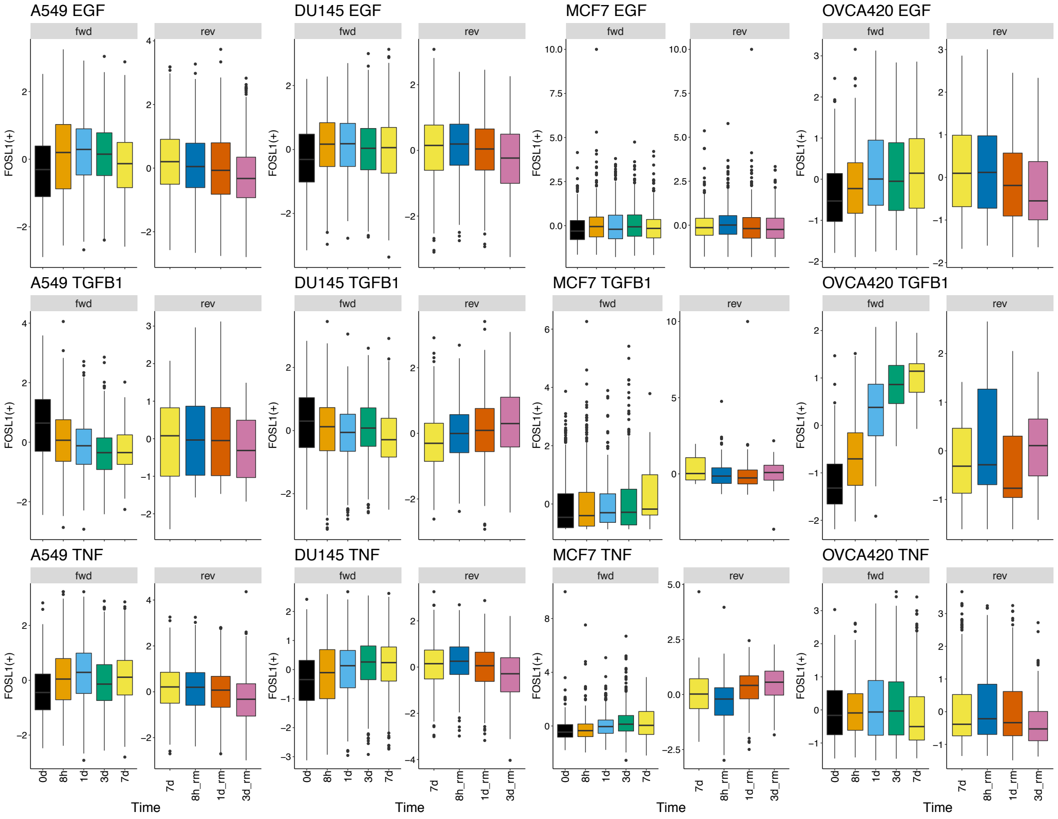
A549 EGF
DU145 EGF
MCF7 EGF
OVCA420 EGF
A549 TGFB1
DU145 TGFB1
MCF7 TGFB1
OVCA420 TGFB1
A549 TNF
DU145 TNF
MCF7 TNF
OVCA420 TNF
fwd
rev
FOSL1(+)
0d
8h
1d
3d
7d
8h_rm
1d_rm
3d_rm
Time

L

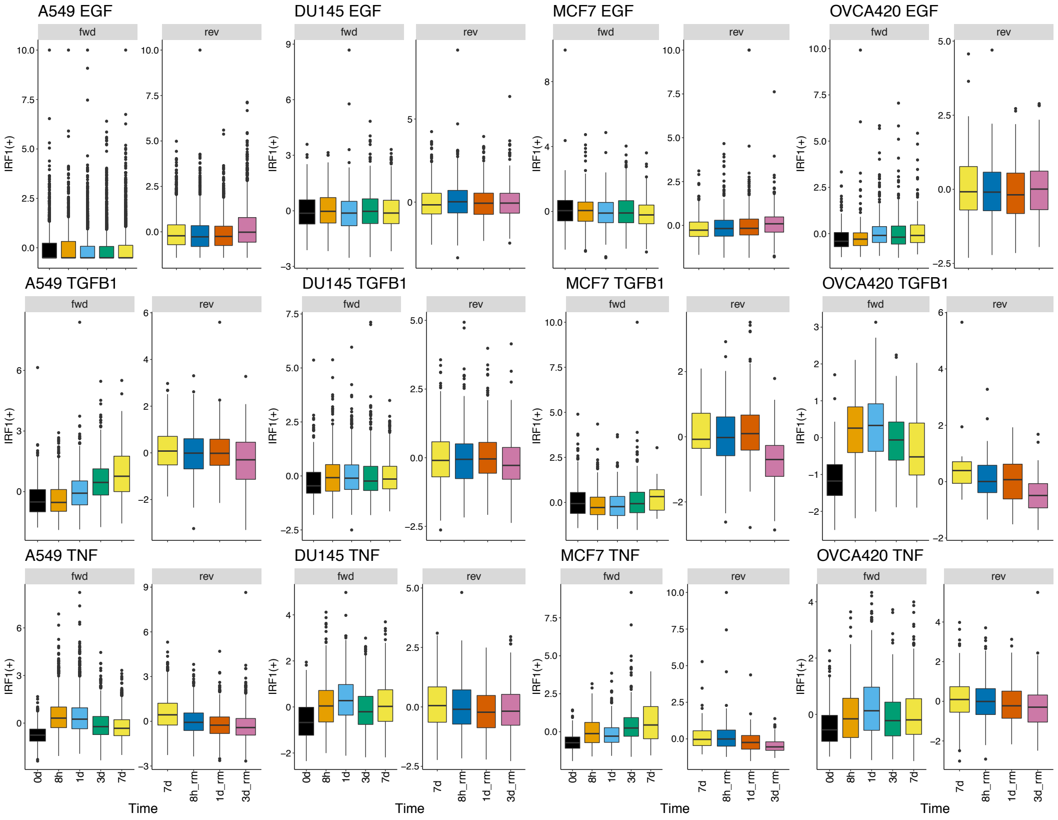
A549 EGF
DU145 EGF
MCF7 EGF
OVCA420 EGF
A549 TGFB1
DU145 TGFB1
MCF7 TGFB1
OVCA420 TGFB1
A549 TNF
DU145 TNF
MCF7 TNF
OVCA420 TNF
fwd
rev
IRF1(+)
0d
8h
1d
3d
7d
8h_rm
1d_rm
3d_rm
Time

M

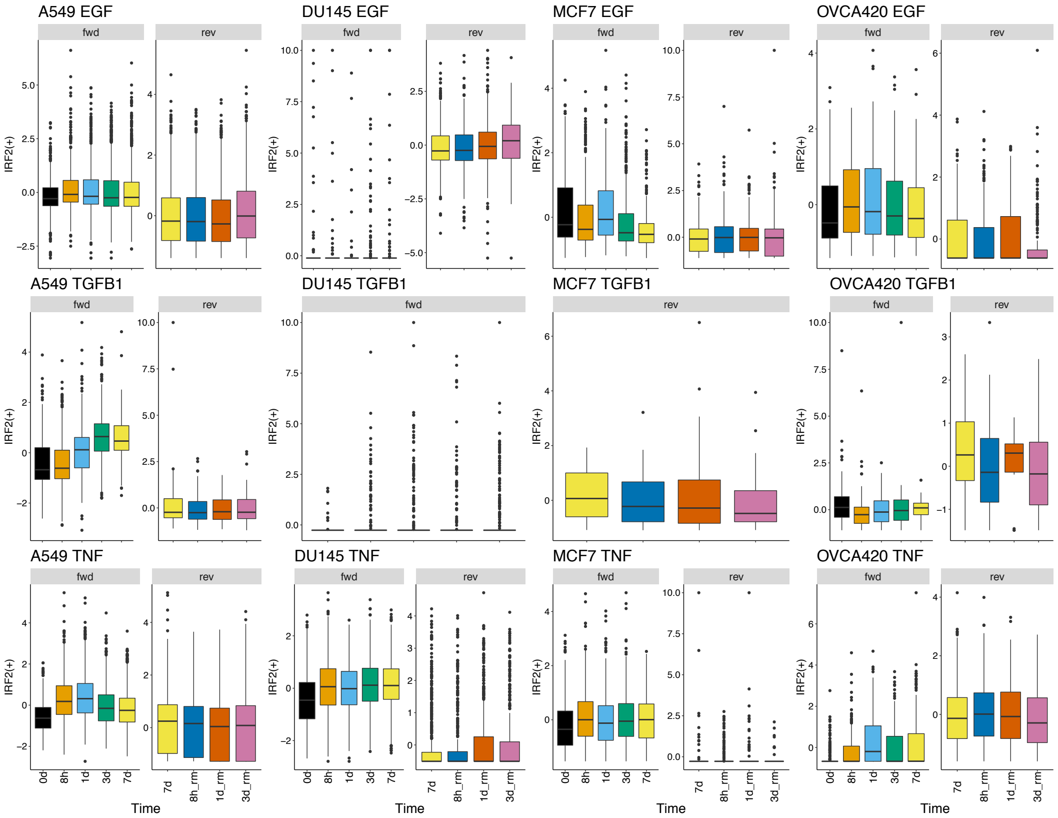

A549 EGF
DU145 EGF
MCF7 EGF
OVCA420 EGF
A549 TGFB1
DU145 TGFB1
MCF7 TGFB1
OVCA420 TGFB1
A549 TNF
DU145 TNF
MCF7 TNF
OVCA420 TNF
fwd
rev
IRF2(+)
0d
8h
1d
3d
7d
7d
8h_rm
1d_rm
3d_rm
Time

N

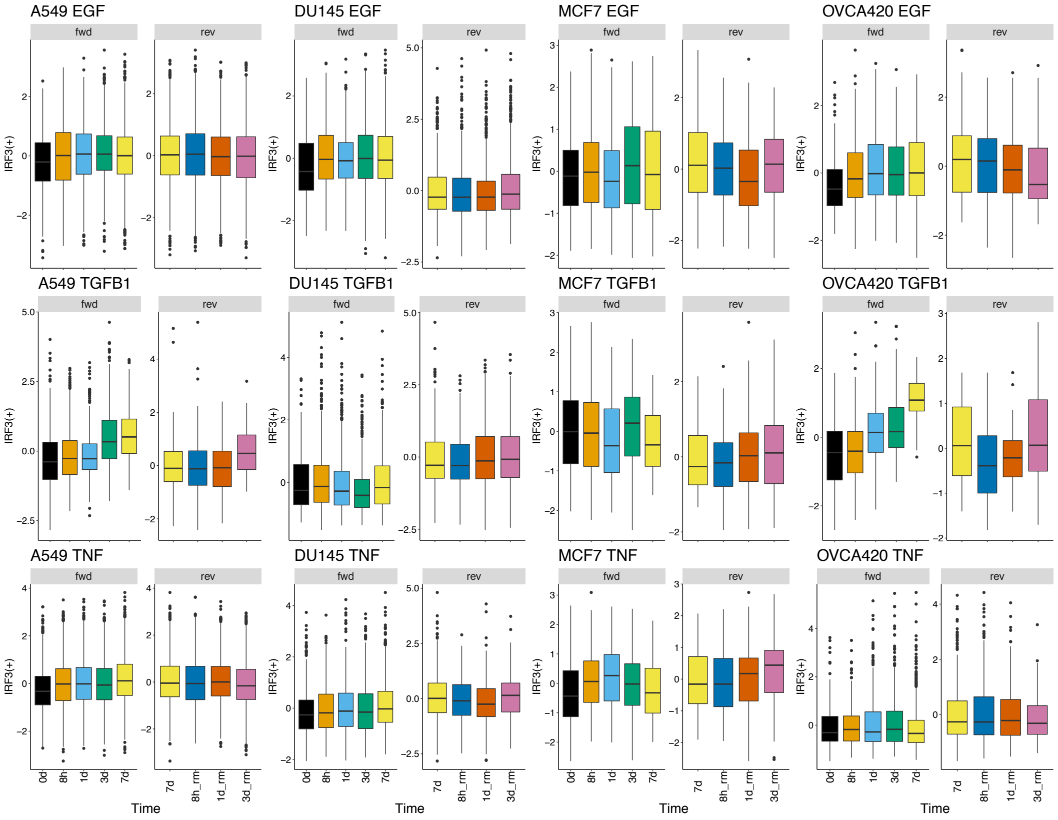

A549 EGF
DU145 EGF
MCF7 EGF
OVCA420 EGF
A549 TGFB1
DU145 TGFB1
MCF7 TGFB1
OVCA420 TGFB1
A549 TNF
DU145 TNF
MCF7 TNF
OVCA420 TNF
fwd
rev
IRF3(+)
0d
8h
1d
3d
7d
7d
8h_rm
1d_rm
3d_rm
Time

O

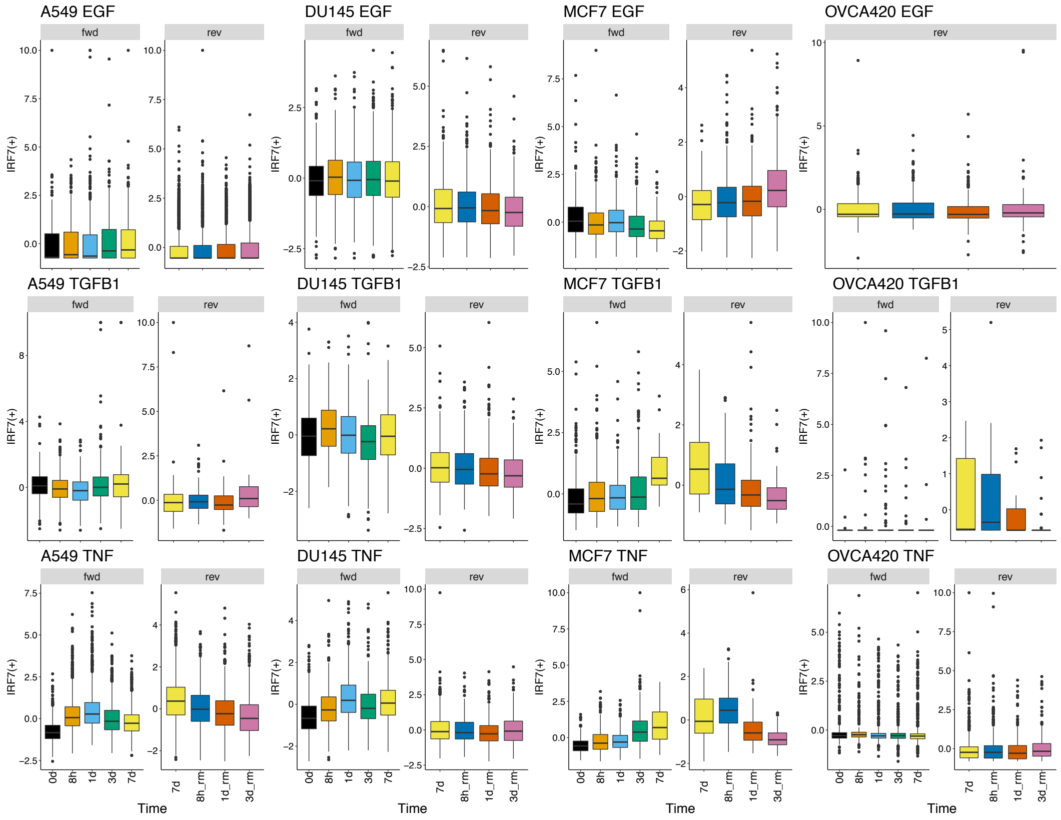
A549 EGF
DU145 EGF
MCF7 EGF
OVCA420 EGF
A549 TGFB1
DU145 TGFB1
MCF7 TGFB1
OVCA420 TGFB1
A549 TNF
DU145 TNF
MCF7 TNF
OVCA420 TNF
fwd
rev
IRF7(+)
0d
8h
1d
3d
7d
7d
8h_rm
1d_rm
3d_rm
Time

P

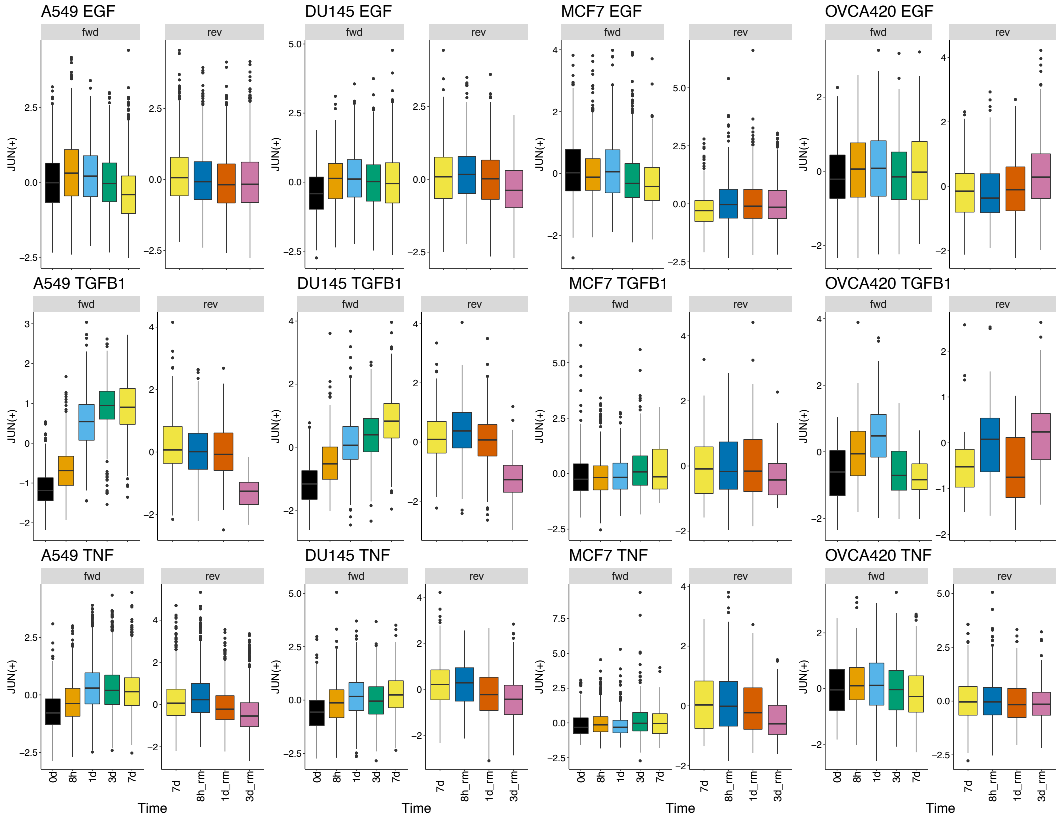
A549 EGF
DU145 EGF
MCF7 EGF
OVCA420 EGF
A549 TGFB1
DU145 TGFB1
MCF7 TGFB1
OVCA420 TGFB1
A549 TNF
DU145 TNF
MCF7 TNF
OVCA420 TNF
fwd
rev
JUN(+)
0d
8h
1d
3d
7d
7d
8h_rm
1d_rm
3d_rm
Time

Q

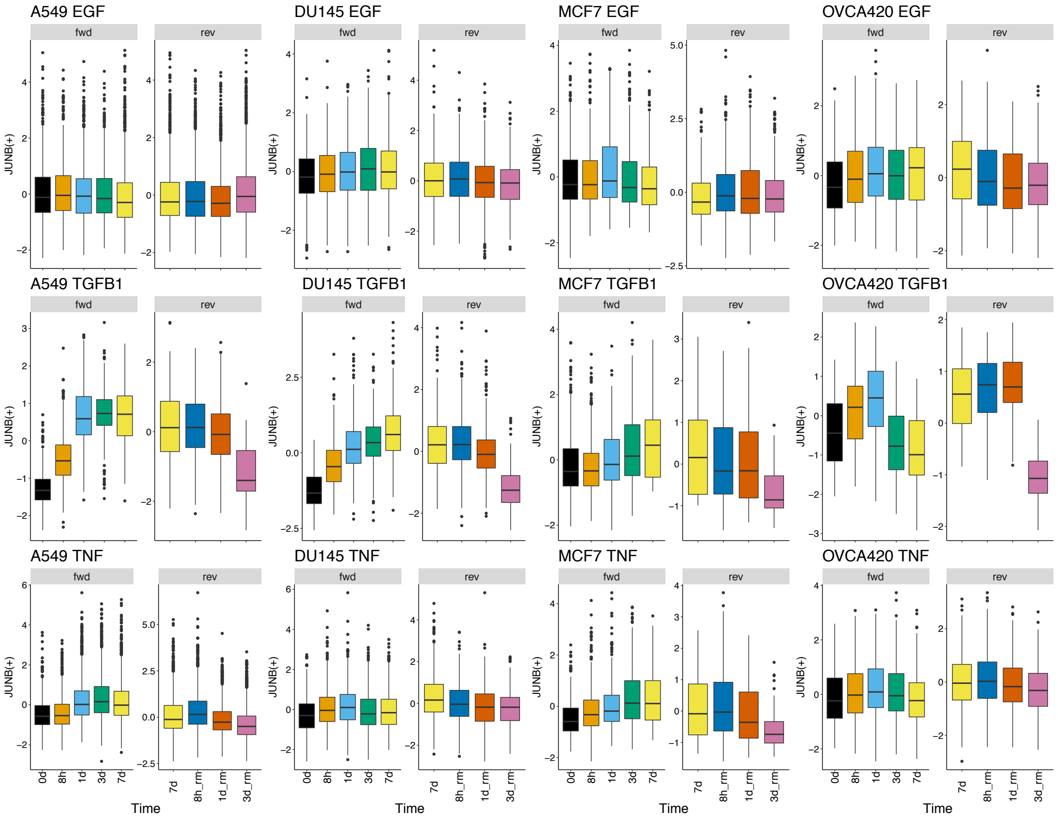
A549 EGF
DU145 EGF
MCF7 EGF
OVCA420 EGF
A549 TGFB1
DU145 TGFB1
MCF7 TGFB1
OVCA420 TGFB1
A549 TNF
DU145 TNF
MCF7 TNF
OVCA420 TNF
fwd
rev
JUNB(+)
0d
8h
1d
3d
7d
7d
8h_rm
1d_rm
3d_rm
Time

R

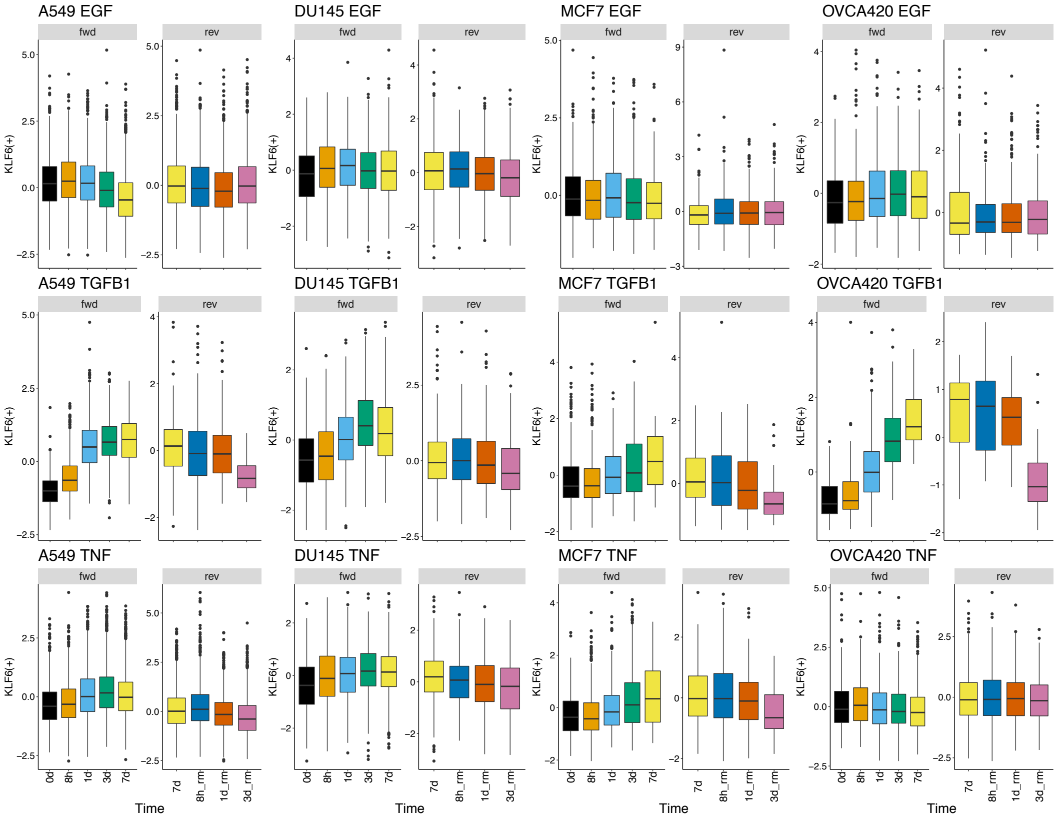
A549 EGF
DU145 EGF
MCF7 EGF
OVCA420 EGF
A549 TGFB1
DU145 TGFB1
MCF7 TGFB1
OVCA420 TGFB1
A549 TNF
DU145 TNF
MCF7 TNF
OVCA420 TNF
fwd
rev
KLF6(+)
0d
8h
1d
3d
7d
7d
8h_rm
1d_rm
3d_rm
Time

S

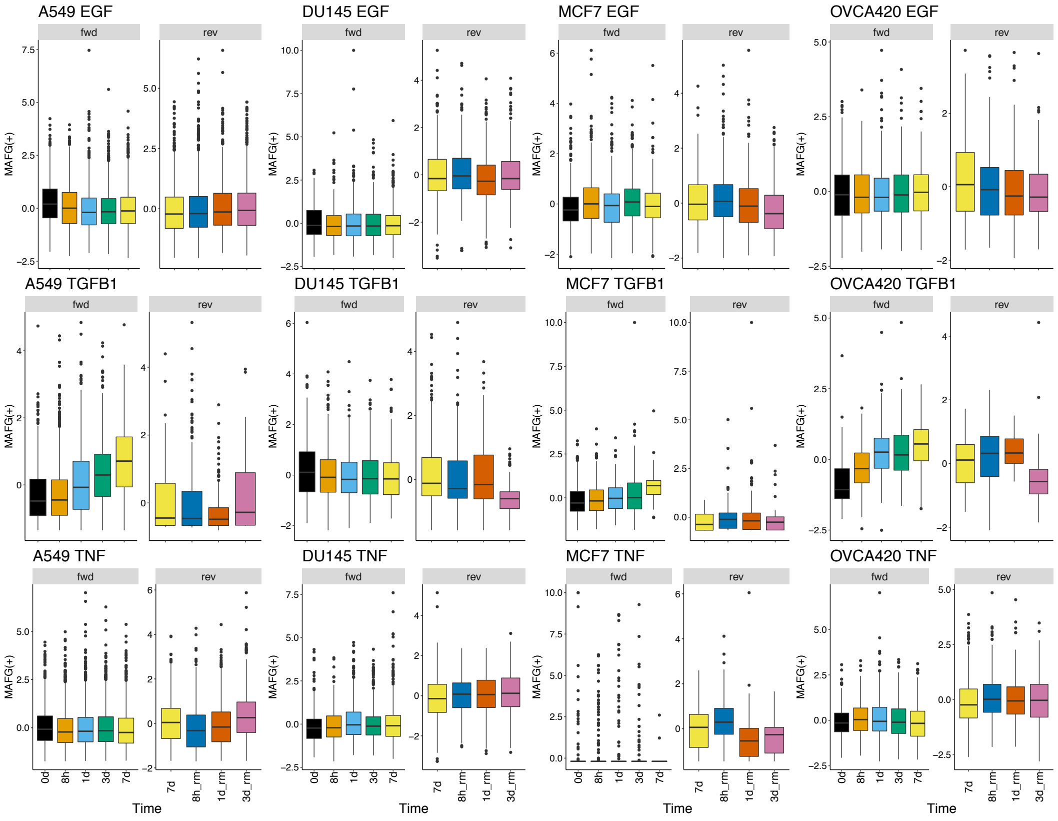

A549 EGF
DU145 EGF
MCF7 EGF
OVCA420 EGF
A549 TGFB1
DU145 TGFB1
MCF7 TGFB1
OVCA420 TGFB1
A549 TNF
DU145 TNF
MCF7 TNF
OVCA420 TNF
fwd
rev
MAFG(+)
0d
8h
1d
3d
7d
7d
8h_rm
1d_rm
3d_rm
Time

T

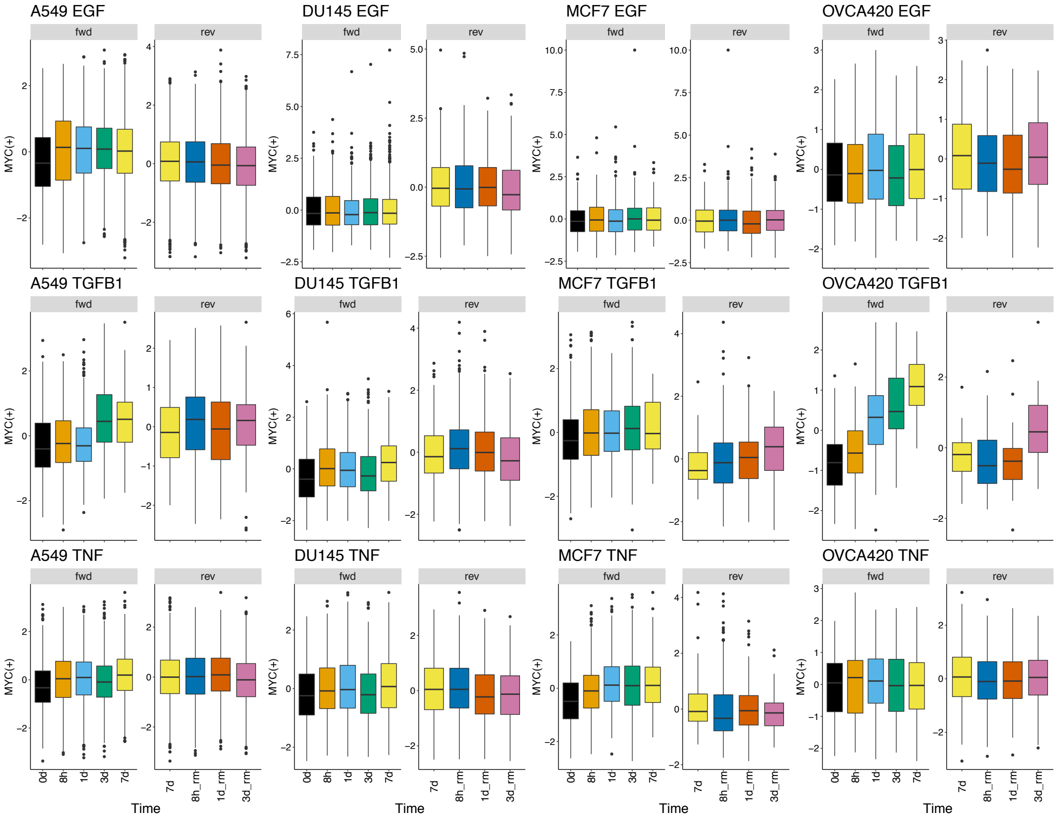

A549 EGF
DU145 EGF
MCF7 EGF
OVCA420 EGF
A549 TGFB1
DU145 TGFB1
MCF7 TGFB1
OVCA420 TGFB1
A549 TNF
DU145 TNF
MCF7 TNF
OVCA420 TNF
fwd
rev
MYC(+)
0d
8h
1d
3d
7d
7d
8h_rm
1d_rm
3d_rm
Time

U

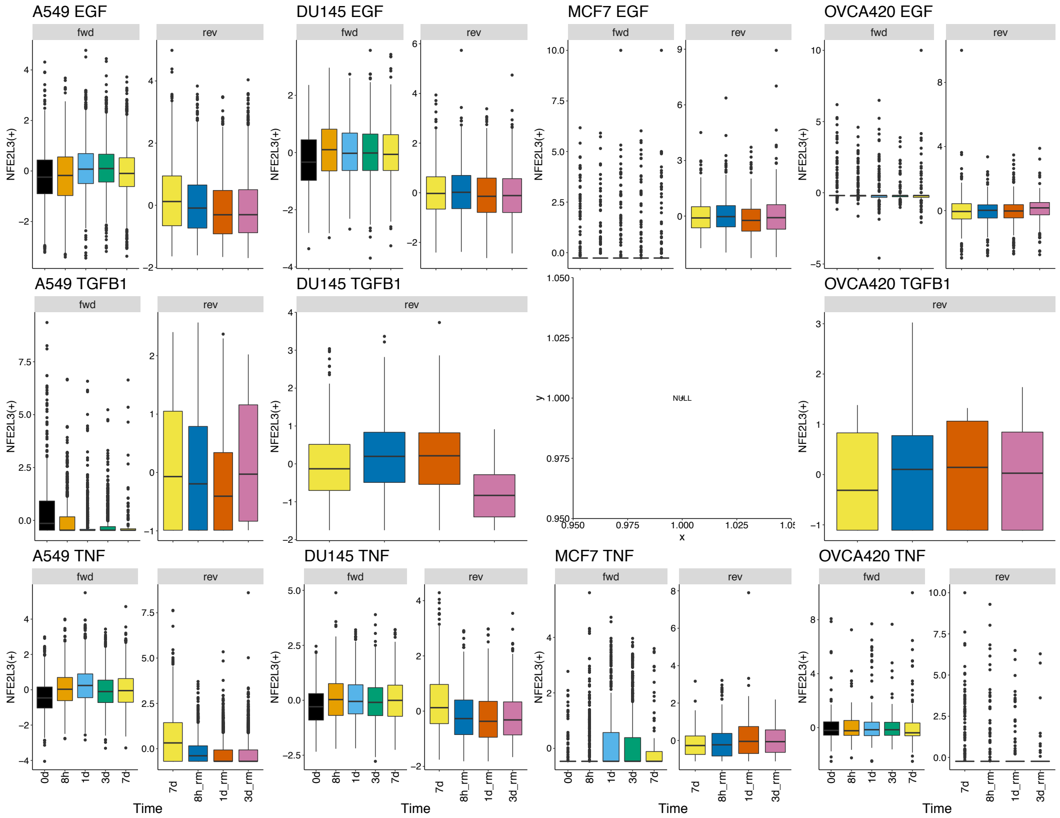
A549 EGF
DU145 EGF
MCF7 EGF
OVCA420 EGF
A549 TGFB1
DU145 TGFB1
OVCA420 TGFB1
A549 TNF
DU145 TNF
MCF7 TNF
OVCA420 TNF
fwd
rev
NFE2L3(+)
NULL
x
y
0d
8h
1d
3d
7d
8h_rm
1d_rm
3d_rm
Time

V

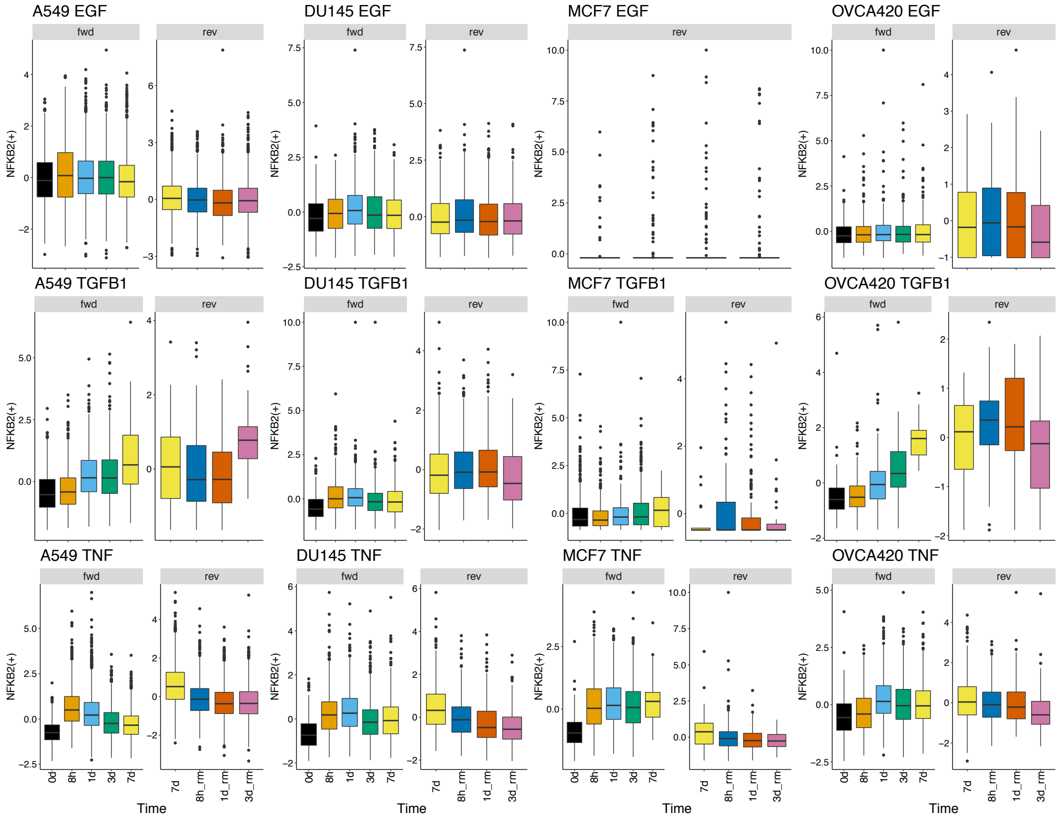
A549 EGF
DU145 EGF
MCF7 EGF
OVCA420 EGF
A549 TGFB1
DU145 TGFB1
MCF7 TGFB1
OVCA420 TGFB1
A549 TNF
DU145 TNF
MCF7 TNF
OVCA420 TNF
fwd
rev
NFKB2(+)
0d
8h
1d
3d
7d
8h_rm
1d_rm
3d_rm
Time

W

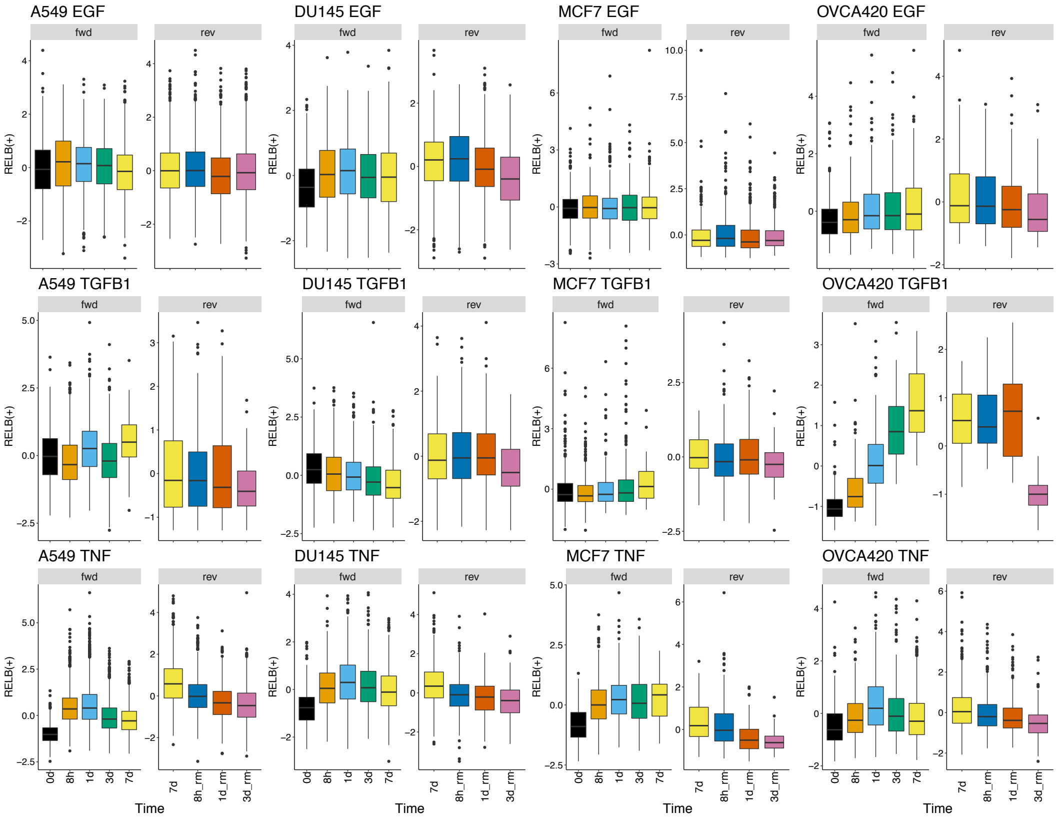

A549 EGF
DU145 EGF
MCF7 EGF
OVCA420 EGF
A549 TGFB1
DU145 TGFB1
MCF7 TGFB1
OVCA420 TGFB1
A549 TNF
DU145 TNF
MCF7 TNF
OVCA420 TNF
fwd
rev
RELB(+)
0d
8h
1d
3d
7d
8h_rm
1d_rm
3d_rm
Time

X

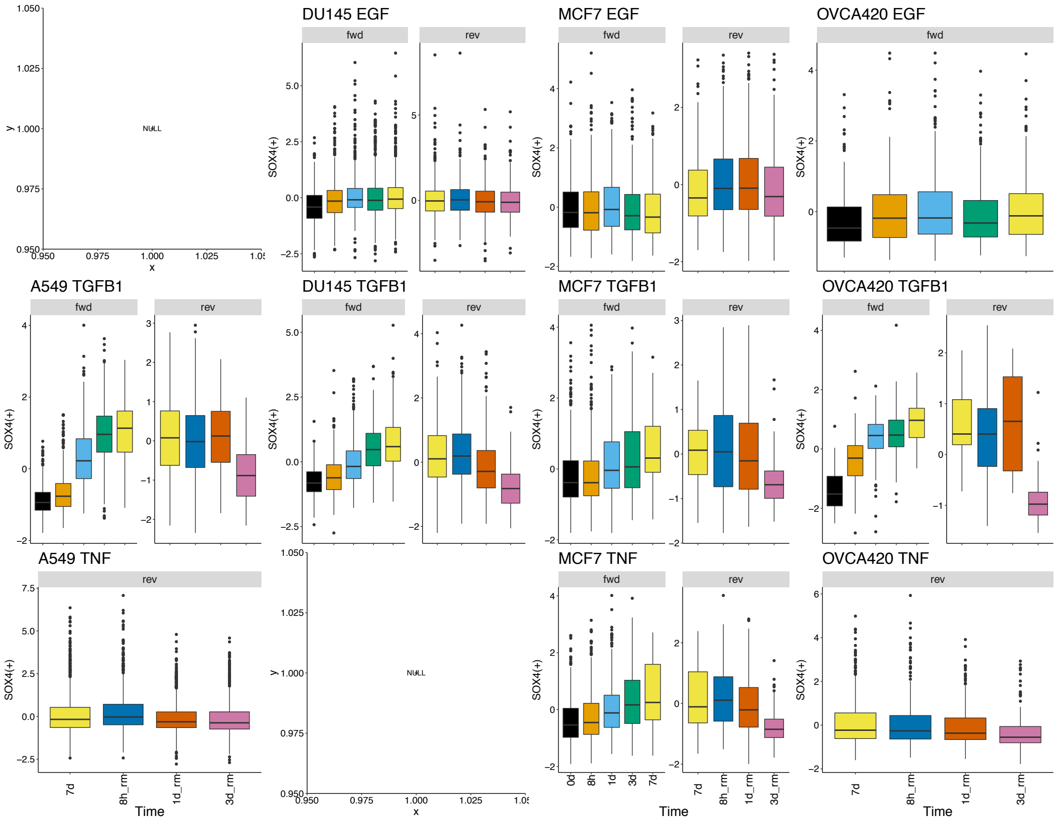

NULL
DU145 EGF
MCF7 EGF
OVCA420 EGF
A549 TGFB1
DU145 TGFB1
MCF7 TGFB1
OVCA420 TGFB1
A549 TNF
MCF7 TNF
OVCA420 TNF
fwd
rev
SOX4(+)
y
x
0d
8h
1d
3d
7d
8h_rm
1d_rm
3d_rm
Time

Y

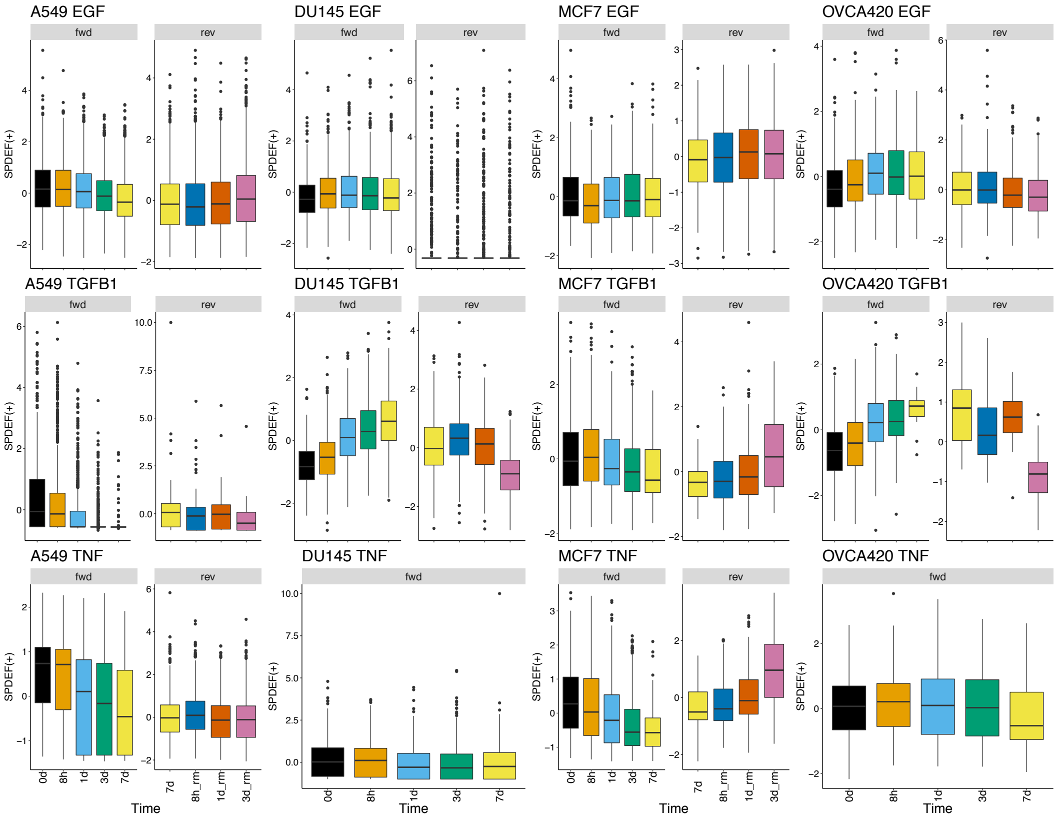
A549 EGF
DU145 EGF
MCF7 EGF
OVCA420 EGF
A549 TGFB1
DU145 TGFB1
MCF7 TGFB1
OVCA420 TGFB1
A549 TNF
DU145 TNF
MCF7 TNF
OVCA420 TNF
fwd
rev
SPDEF(+)
0d
8h
1d
3d
7d
8h_rm
1d_rm
3d_rm
Time

Z

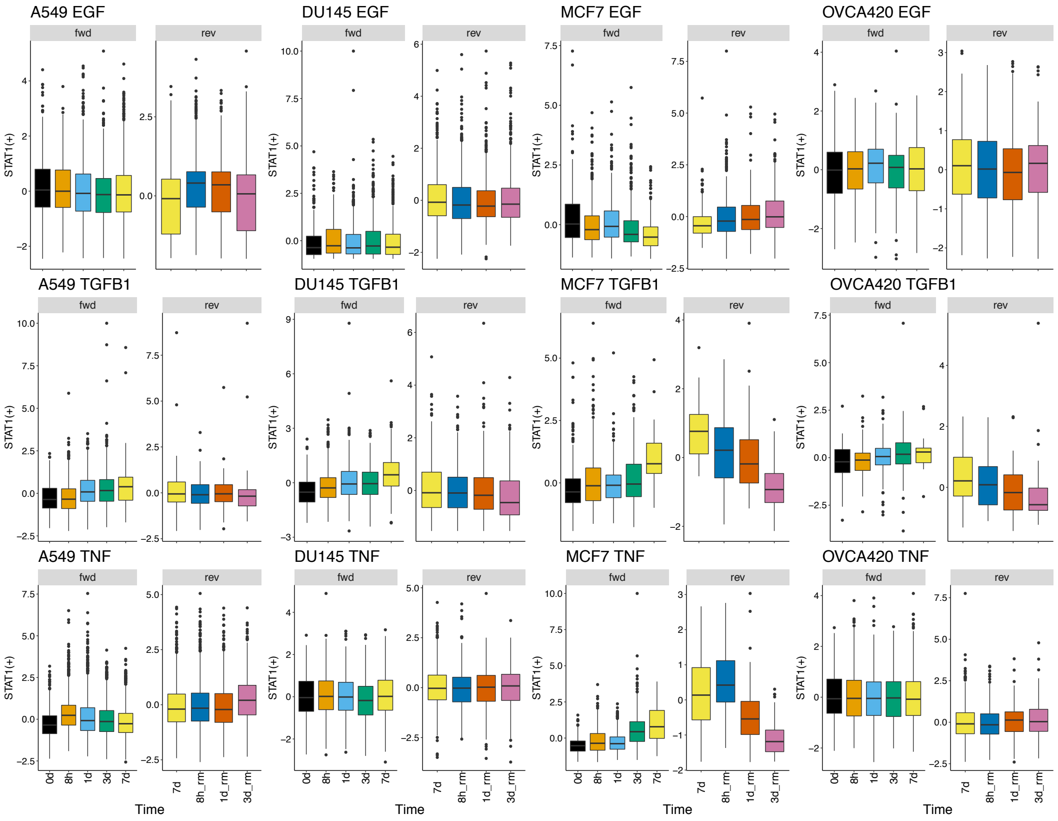
A549 EGF
DU145 EGF
MCF7 EGF
OVCA420 EGF
A549 TGFB1
DU145 TGFB1
MCF7 TGFB1
OVCA420 TGFB1
A549 TNF
DU145 TNF
MCF7 TNF
OVCA420 TNF
fwd
rev
STAT1(+)
0d
8h
1d
3d
7d
8h_rm
1d_rm
3d_rm
Time

AA

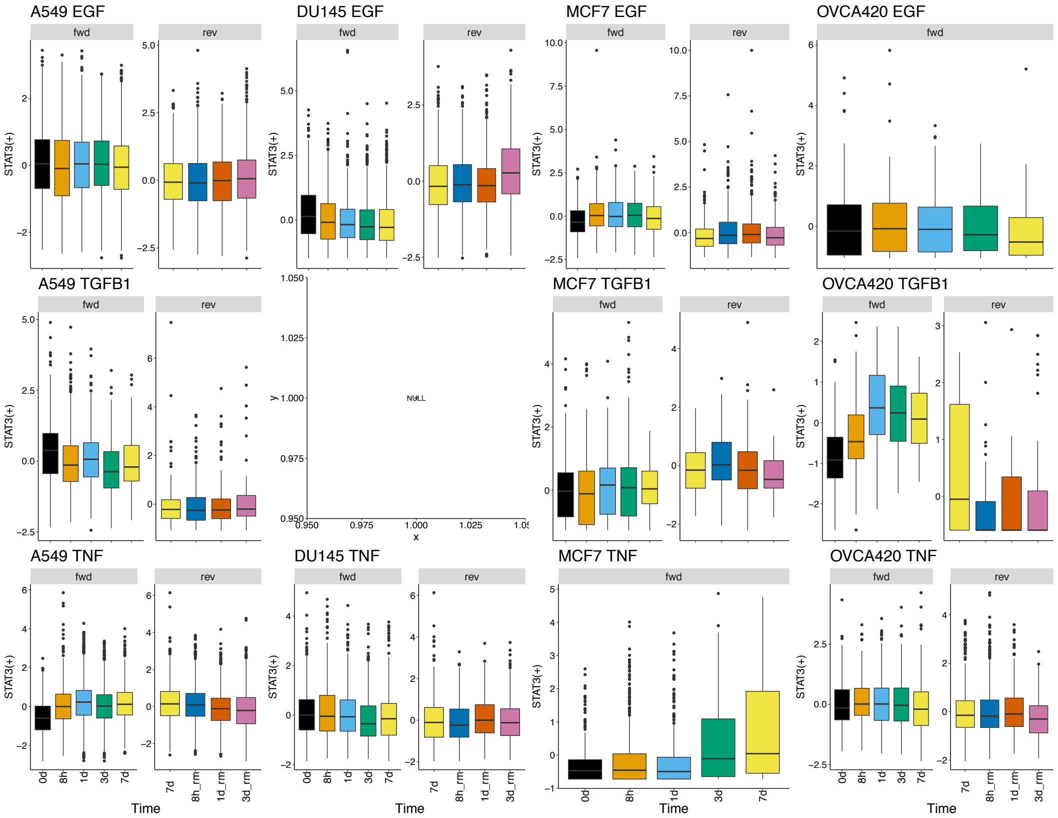
A549 EGF
DU145 EGF
MCF7 EGF
OVCA420 EGF
A549 TGFB1
MCF7 TGFB1
OVCA420 TGFB1
A549 TNF
DU145 TNF
MCF7 TNF
OVCA420 TNF
fwd
rev
STAT3(+)
NULL
y
x
0d
8h
1d
3d
7d
8h_rm
1d_rm
3d_rm
Time

AB

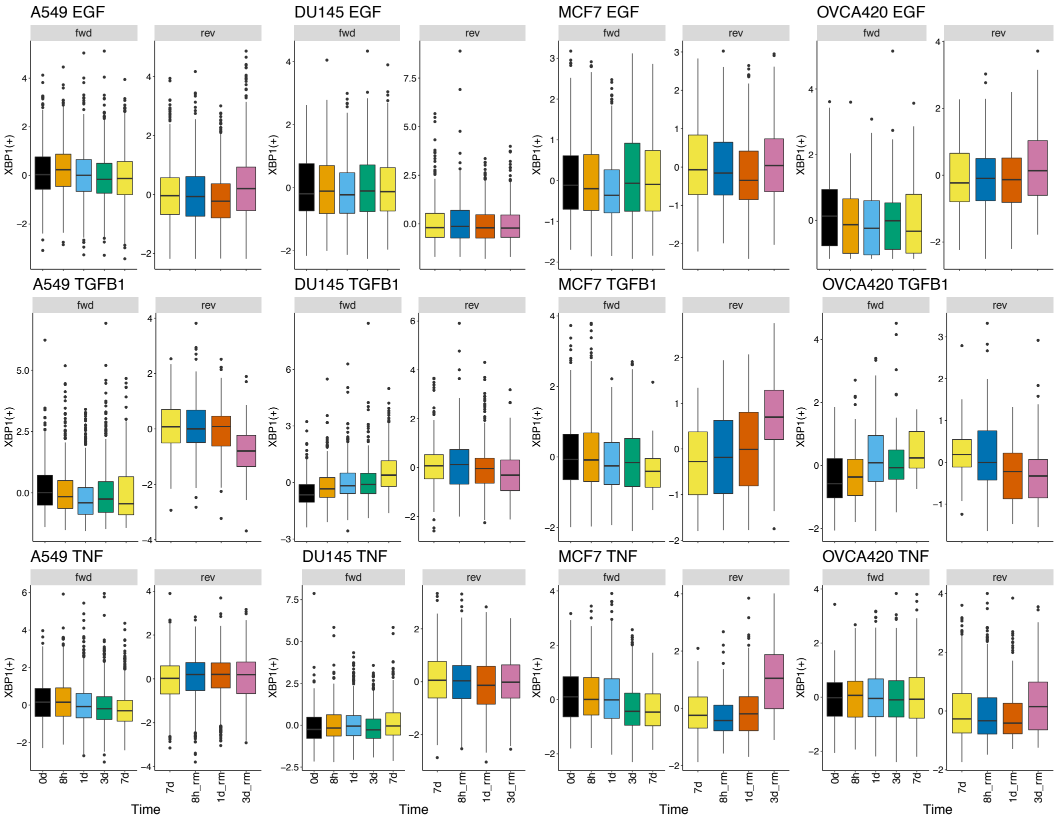
A549 EGF
DU145 EGF
MCF7 EGF
OVCA420 EGF
A549 TGFB1
DU145 TGFB1
MCF7 TGFB1
OVCA420 TGFB1
A549 TNF
DU145 TNF
MCF7 TNF
OVCA420 TNF
fwd
rev
XBP1(+)
0d
8h
1d
3d
7d
8h_rm
1d_rm
3d_rm
Time

Figure S1A-AB. Activity profiles over time of 28 highly conserved TFs across all experimental conditions. Activity values shown are calculated from SCENIC. Box and whisker plots show the middle 50% of activity values within the box with a line indicating the mean value. Whiskers show the top and bottom quartiles and individual points are statistical outliers. Fwd denotes the timepoints after signal induction and rev denotes the timepoints after signal removal. Each panel shows one gene across all 12 experimental conditions. Missing plots indicate that a TF was not identified as a regulon by SCENIC in that experimental dataset.

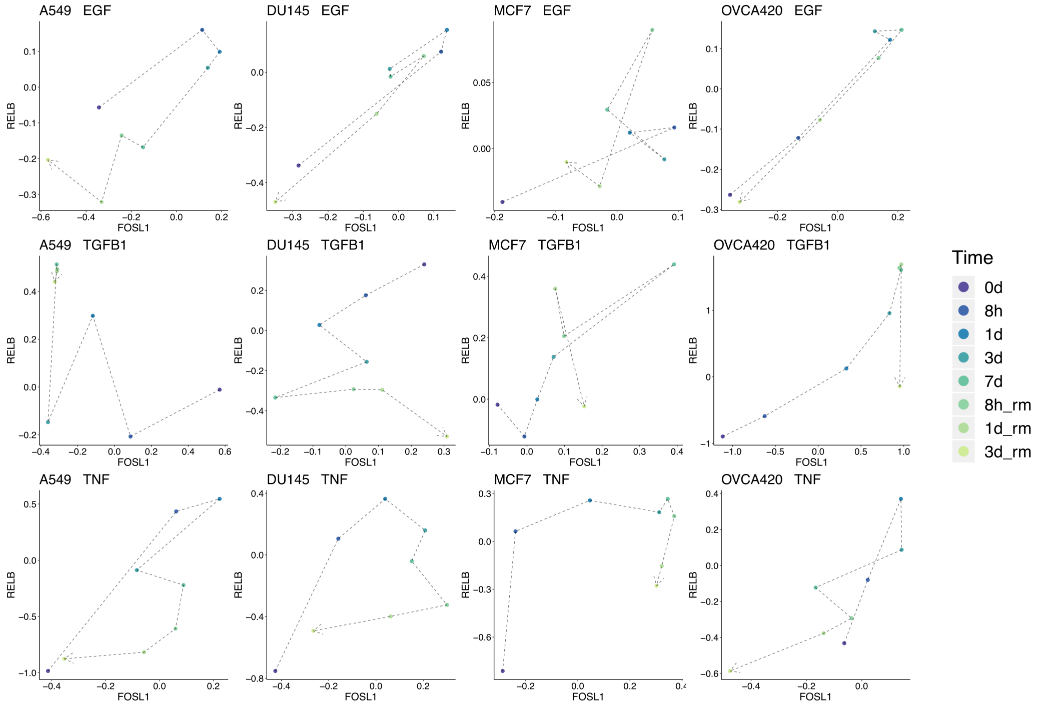

Figure S2. Signal chronology across different conditions for alternate gene pairs. Average TF activity of RELB vs FOSL1 for each timepoint. Data from 7d onward is scaled on a linear model to match the 7d distributions for the forward direction. Point color denotes timepoint and chronology is demonstrated with a dashed arrow.

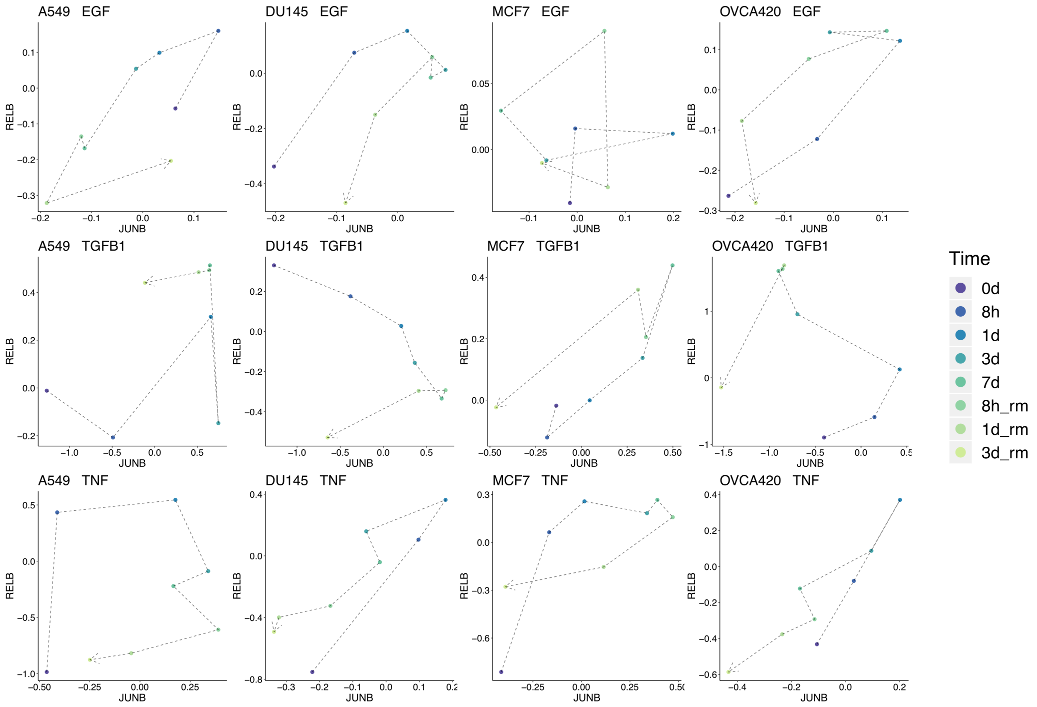

Figure S3. Signal chronology across different conditions for alternate gene pairs. Average TF activity of RELB vs JUNB for each timepoint. Data from 7d onward is scaled on a linear model to match the 7d distributions for the forward direction. Point color denotes timepoint and chronology is demonstrated with a dashed arrow.

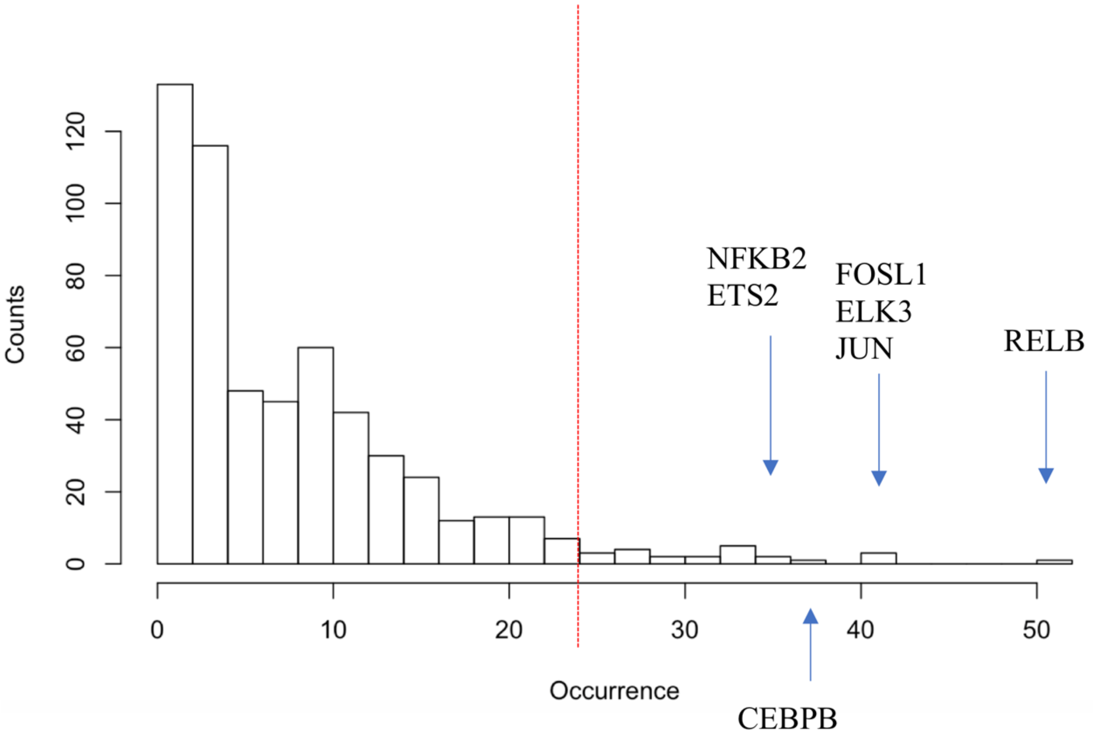

Selected TFs: BHLHE40, CEBPB, CREB3, CREM, ELK3, ESRRA, ETS1, ETS2, FOS, FOSB, FOSL1, IRF1, IRF2, IRF3, IRF7, JUN, JUNB, KLF6, MAFG, MYC, NFE2L3, NFKB2, RELB, SOX4, SPDEF, STAT1, STAT3, XBP1

Figure S4. Number of TFs featuring as differentially active TFs (DATF) out of 84 comparisons. For example, RELB appears as a DATF in 52 comparisons. TFs coming up as DATFs in more than 24 comparisons were selected as common TFs. TF names in red indicate AP-1 and NFKB genes, common downstream targets of the EMT inducing signals.

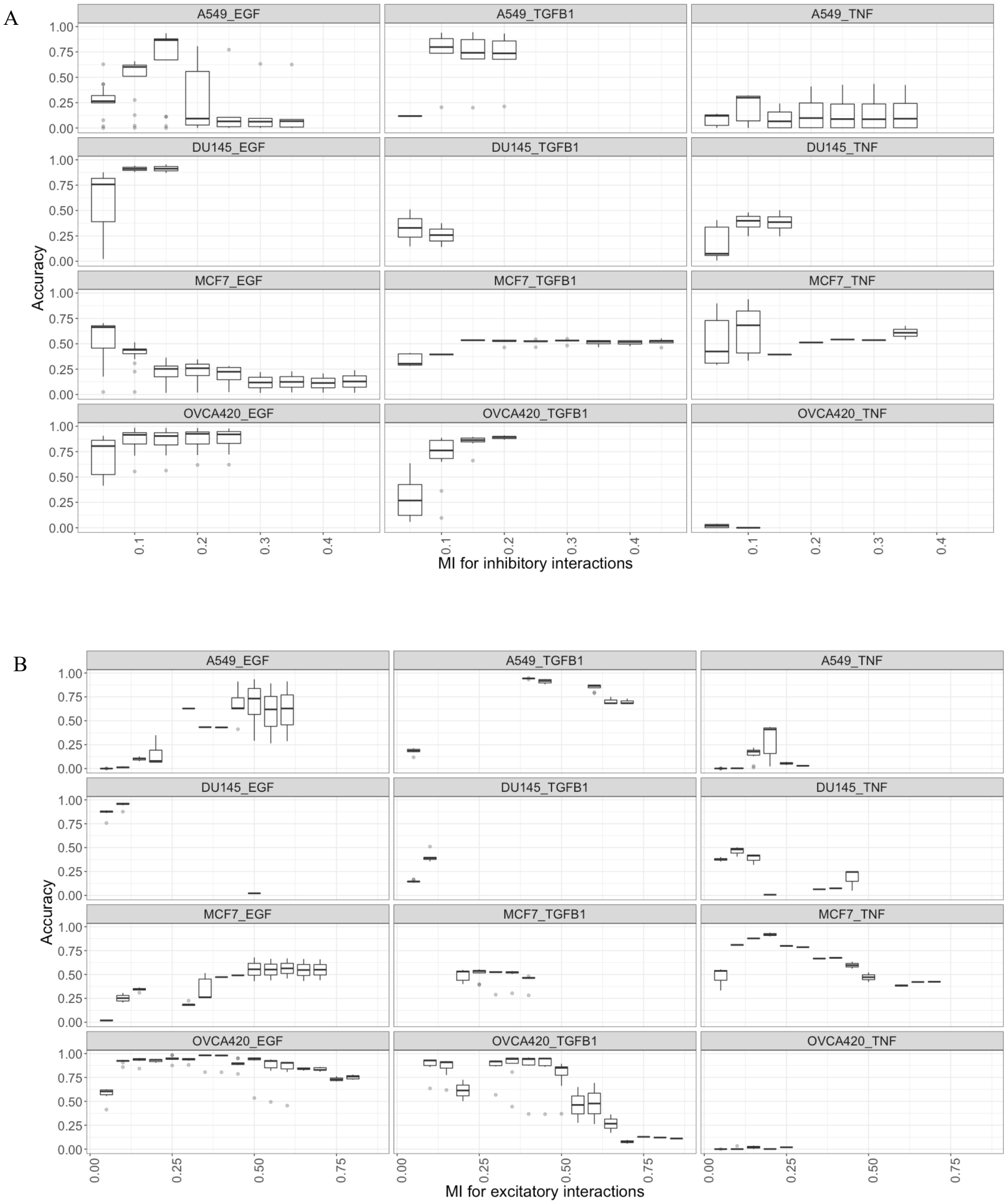
A
A549_EGF
A549_TGFB1
A549_TNF
DU145_EGF
DU145_TGFB1
DU145_TNF
MCF7_EGF
MCF7_TGFB1
MCF7_TNF
OVCA420_EGF
OVCA420_TGFB1
OVCA420_TNF
Accuracy
1.00
0.75
0.50
0.25
0.00
0.1
0.2
0.3
0.4
MI for inhibitory interactions
B
A549_EGF
A549_TGFB1
A549_TNF
DU145_EGF
DU145_TGFB1
DU145_TNF
MCF7_EGF
MCF7_TGFB1
MCF7_TNF
OVCA420_EGF
OVCA420_TGFB1
OVCA420_TNF
Accuracy
1.00
0.75
0.50
0.25
0.00
0.00
0.25
0.50
0.75
MI for excitatory interactions

C

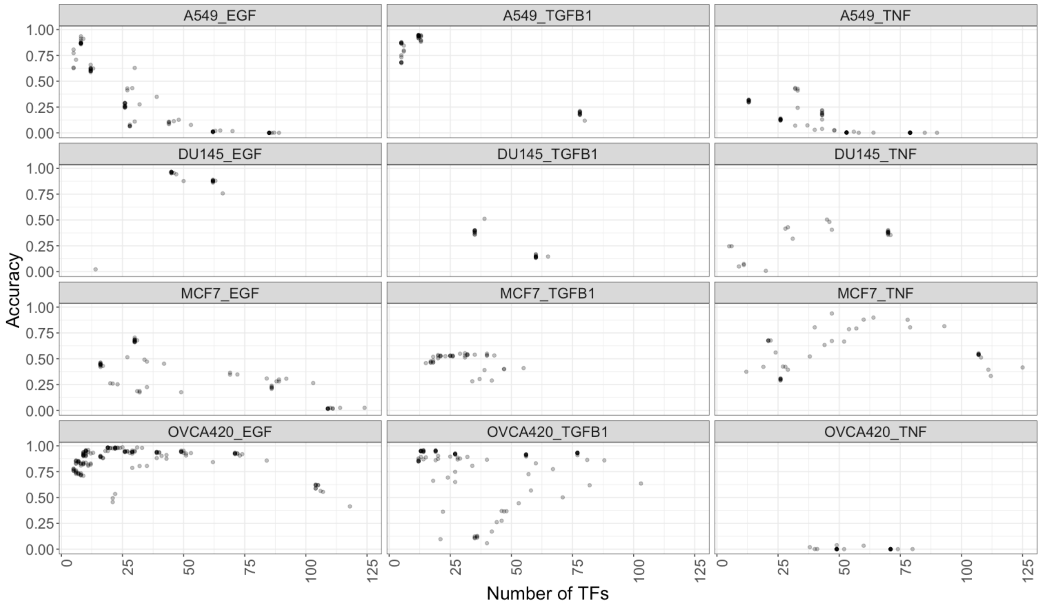

A549_EGF
A549_TGFB1
A549_TNF
DU145_EGF
DU145_TGFB1
DU145_TNF
MCF7_EGF
MCF7_TGFB1
MCF7_TNF
OVCA420_EGF
OVCA420_TGFB1
OVCA420_TNF
Accuracy
Number of TFs

D

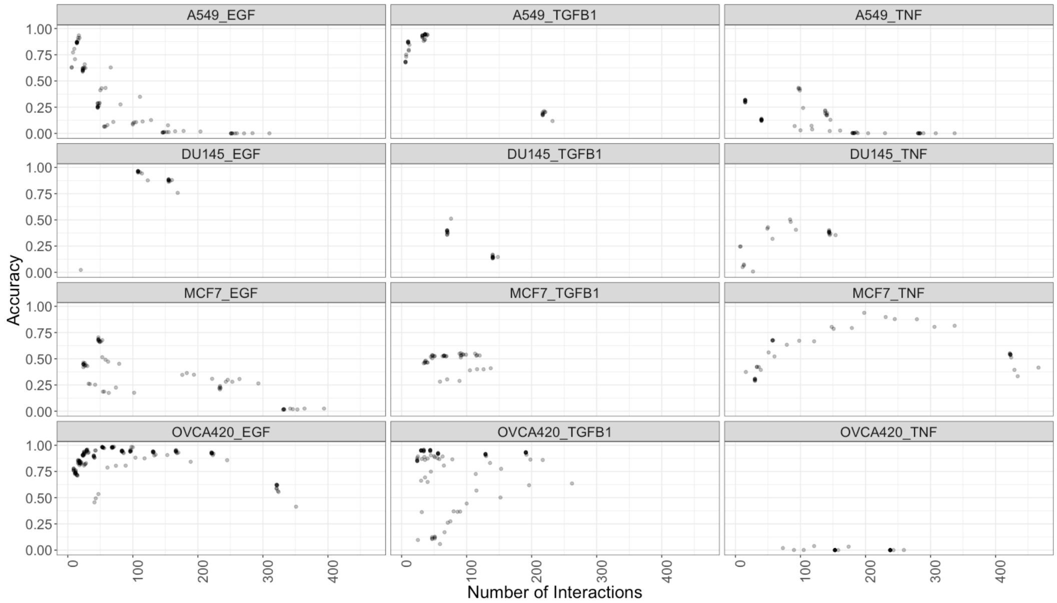

A549_EGF
A549_TGFB1
A549_TNF
DU145_EGF
DU145_TGFB1
DU145_TNF
MCF7_EGF
MCF7_TGFB1
MCF7_TNF
OVCA420_EGF
OVCA420_TGFB1
OVCA420_TNF
Accuracy
Number of Interactions

E

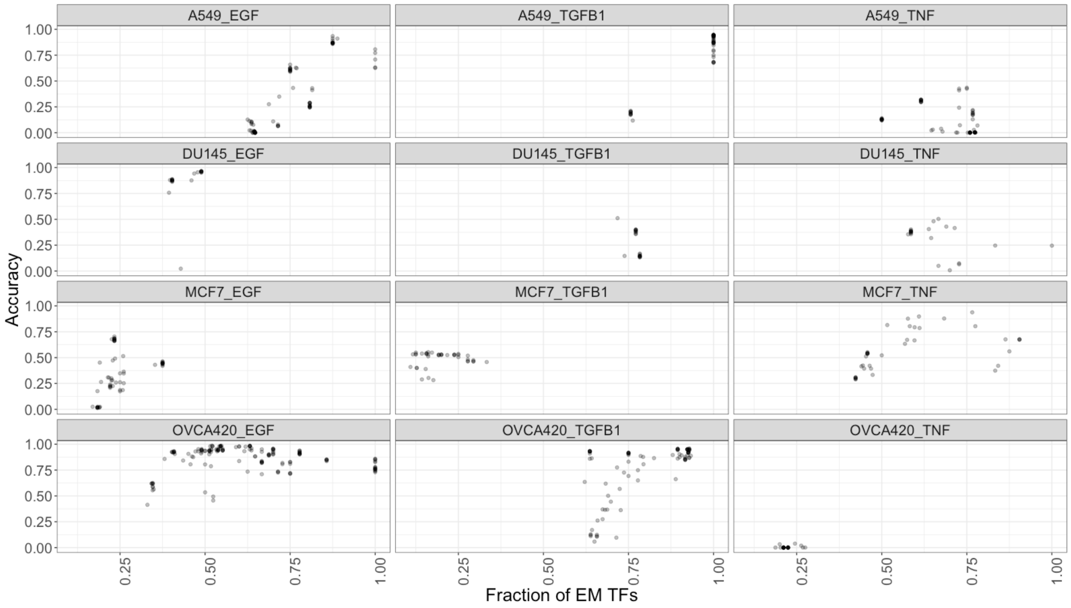

A549_EGF
A549_TGFB1
A549_TNF
DU145_EGF
DU145_TGFB1
DU145_TNF
MCF7_EGF
MCF7_TGFB1
MCF7_TNF
OVCA420_EGF
OVCA420_TGFB1
OVCA420_TNF
Accuracy
Fraction of EM TFs
0.00
0.25
0.50
0.75
1.00

F

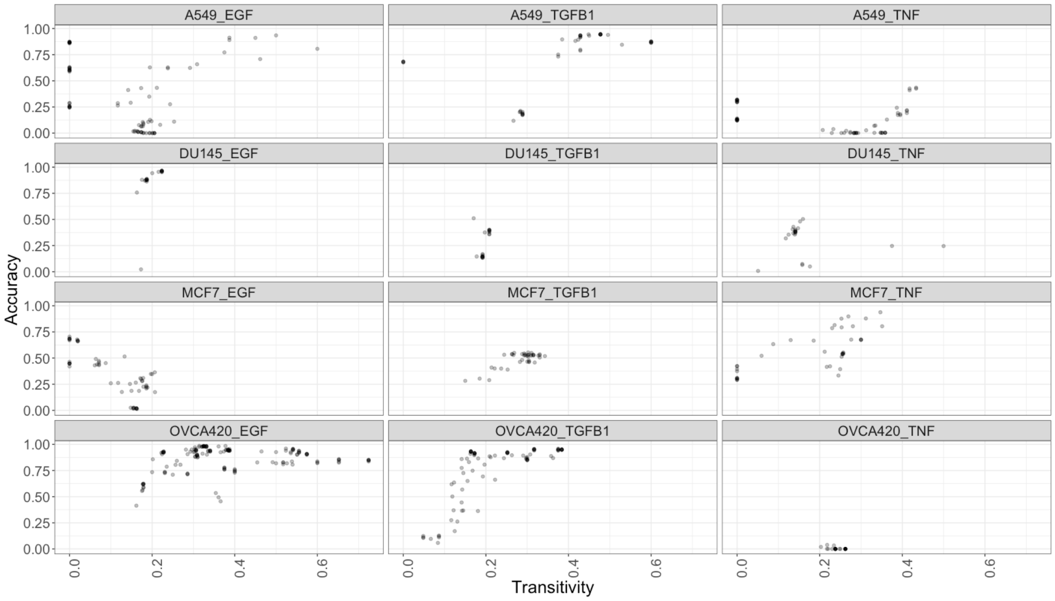

A549_EGF
A549_TGFB1
A549_TNF
DU145_EGF
DU145_TGFB1
DU145_TNF
MCF7_EGF
MCF7_TGFB1
MCF7_TNF
OVCA420_EGF
OVCA420_TGFB1
OVCA420_TNF
Accuracy
Transitivity
0.00
0.25
0.50
0.75
1.00
0.0
0.2
0.4
0.6

G

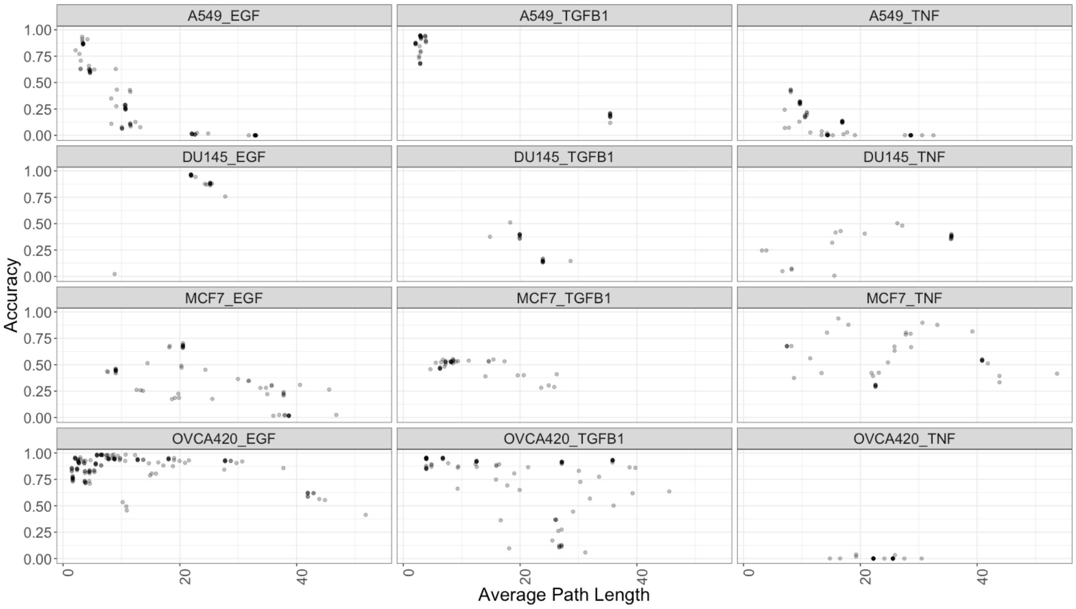

H

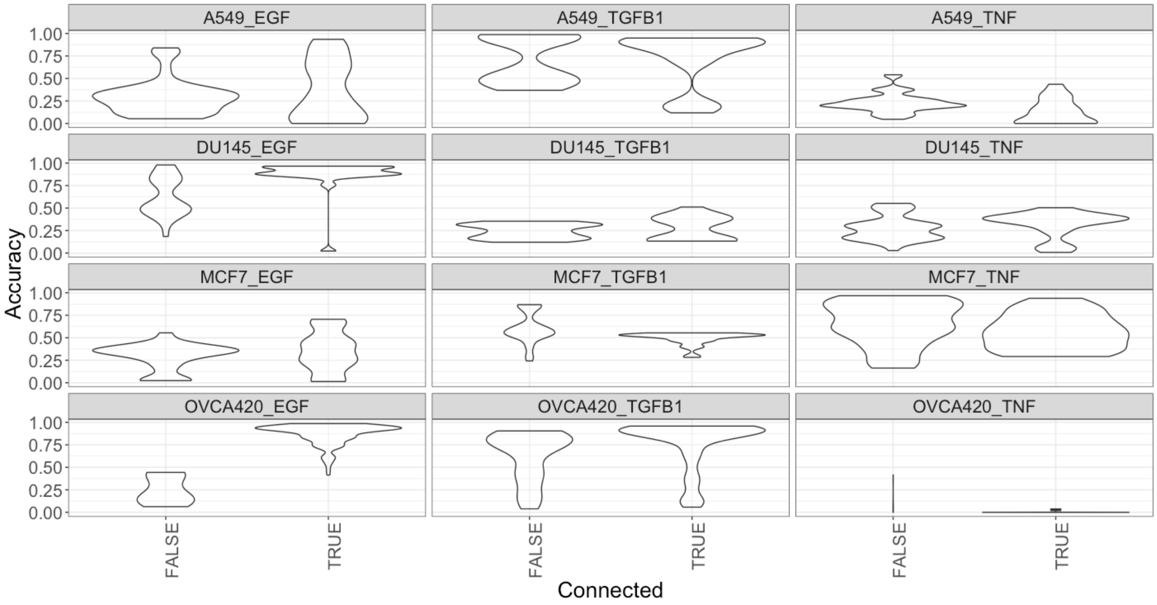

Figure S5. Accuracy dependence on various network properties for each condition. **(A-B)** Box plots showing accuracy of networks for various **(A)** mutual information cutoffs for inhibitory interactions, **(B)** mutual information cutoffs for excitatory interactions. Points showing accuracy (measured as fraction of models that can be classified as E or M) of networks for various **(C)** number of TFs in the network, **(D)** number of interactions in the network, **(E)** TFs assigned as E

or M using experimental data, **(F)** transitivity or clustering coefficient of networks, **(G)** average path length of the networks. **(H)** Violin plots of accuracy values for connected and unconnected networks. Note that only connected networks were used for all analysis in the manuscript.

A Average activity over time for OVCA420 TGFB1

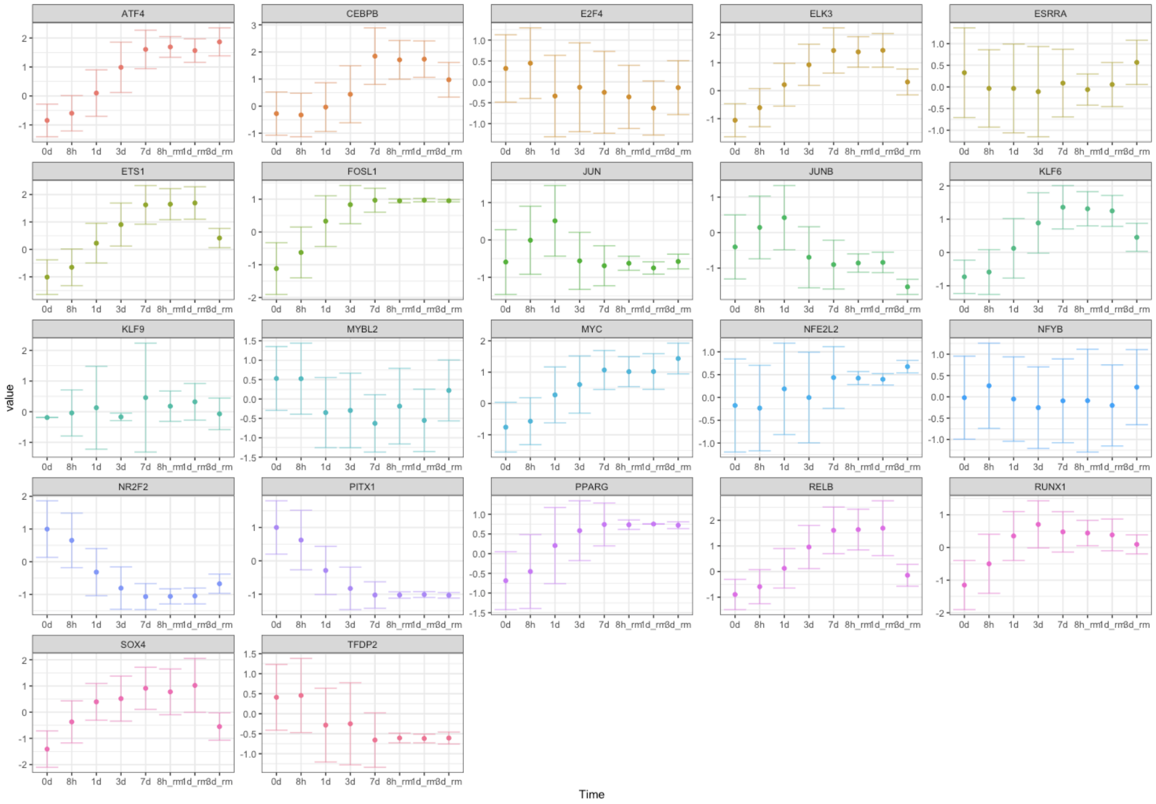

B Average activity over time for OVCA420 TGFB1 network in simulations during signal induction and removal.

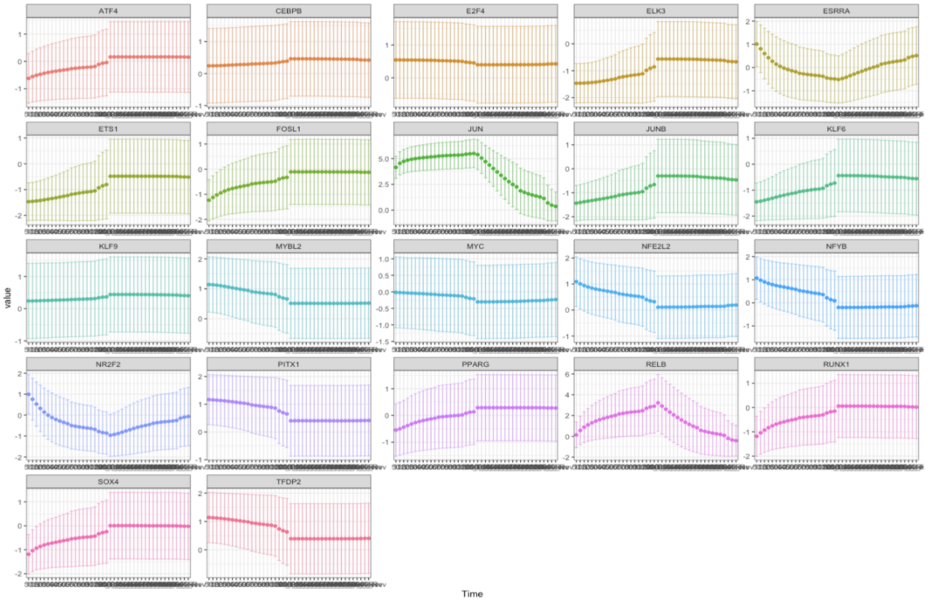

C Average activity over time for OVCA420 TGFB1 network in simulations during signal induction and inhibition.

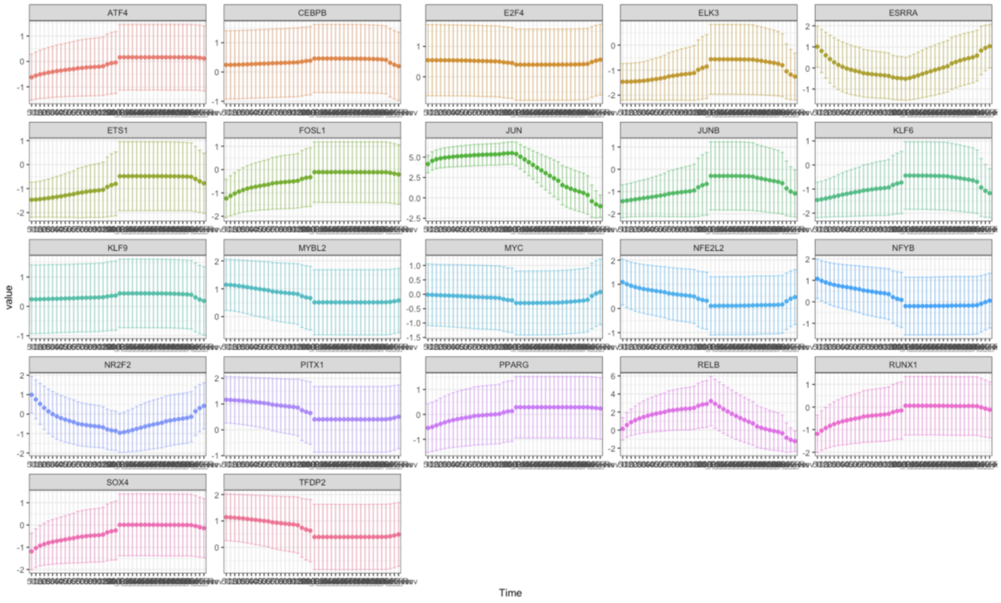

Figure S6. The mean and standard deviation of various TFs in the OVCA420 TGFB1 network at multiple time points in experiments **(A)** and simulations **(B-C)**. The signal was applied till mid point and then removed (B) or inhibited (C). The expressions appear to increase/decrease suddenly at the mid-point as it corresponds to a large jump in time from T=50 to T=2000. Signal was removed or inhibited after T = 2000.

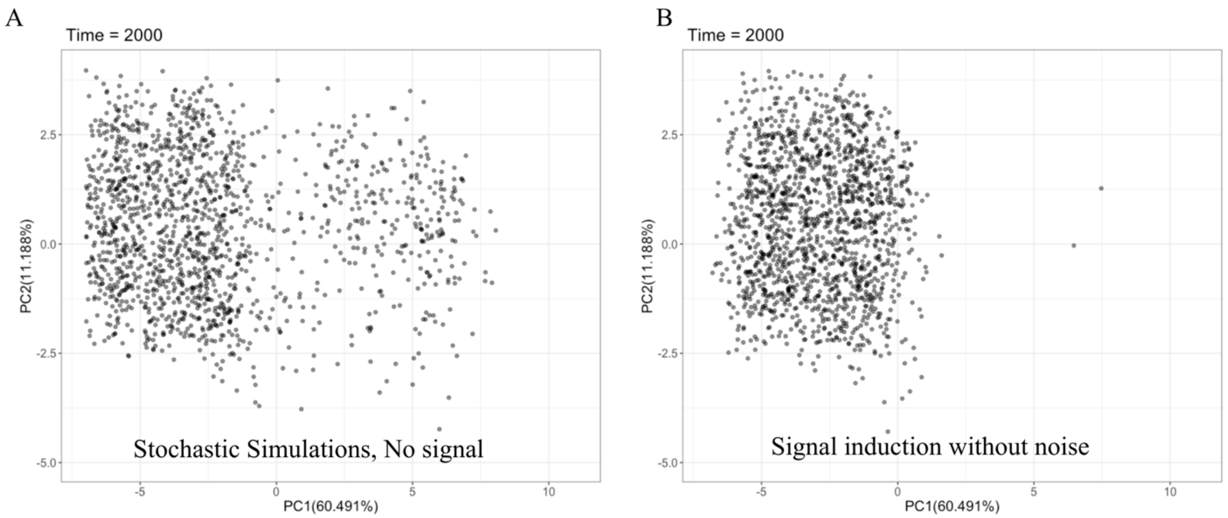

Figure S7. The simulated profiles at T=2000 using only noise **(A)** or only induction **(B)** in E-state models. The simulations indicate that either the noise (0.05 here) or signal induction only is not sufficient to drive state transitions for a substantial number of models. However, much more state transitions can be achieved by adding both noise and signal induction (Figure 7E in main text).

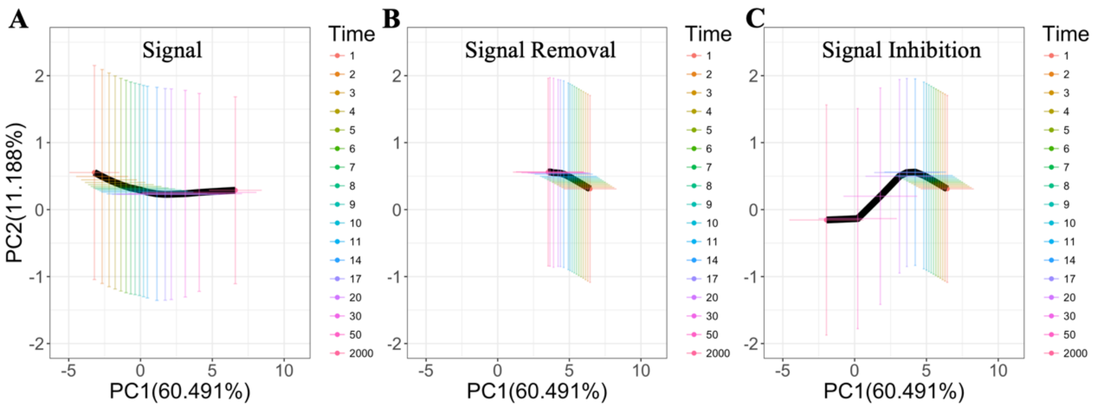

Figure S8. Mean and standard deviation of the simulated profiles of the E-state models that transited to M state during **(A)** signal induction (left), **(B)** removal (middle), and **(C)** inhibition (right) at multiple time points projected on the first two principal components of the simulated data of all models. These models were selected from all of the E models whose PC1 component was larger than 2.5 at the end of signal induction simulations. There are ~42% of E models belonging to this type.

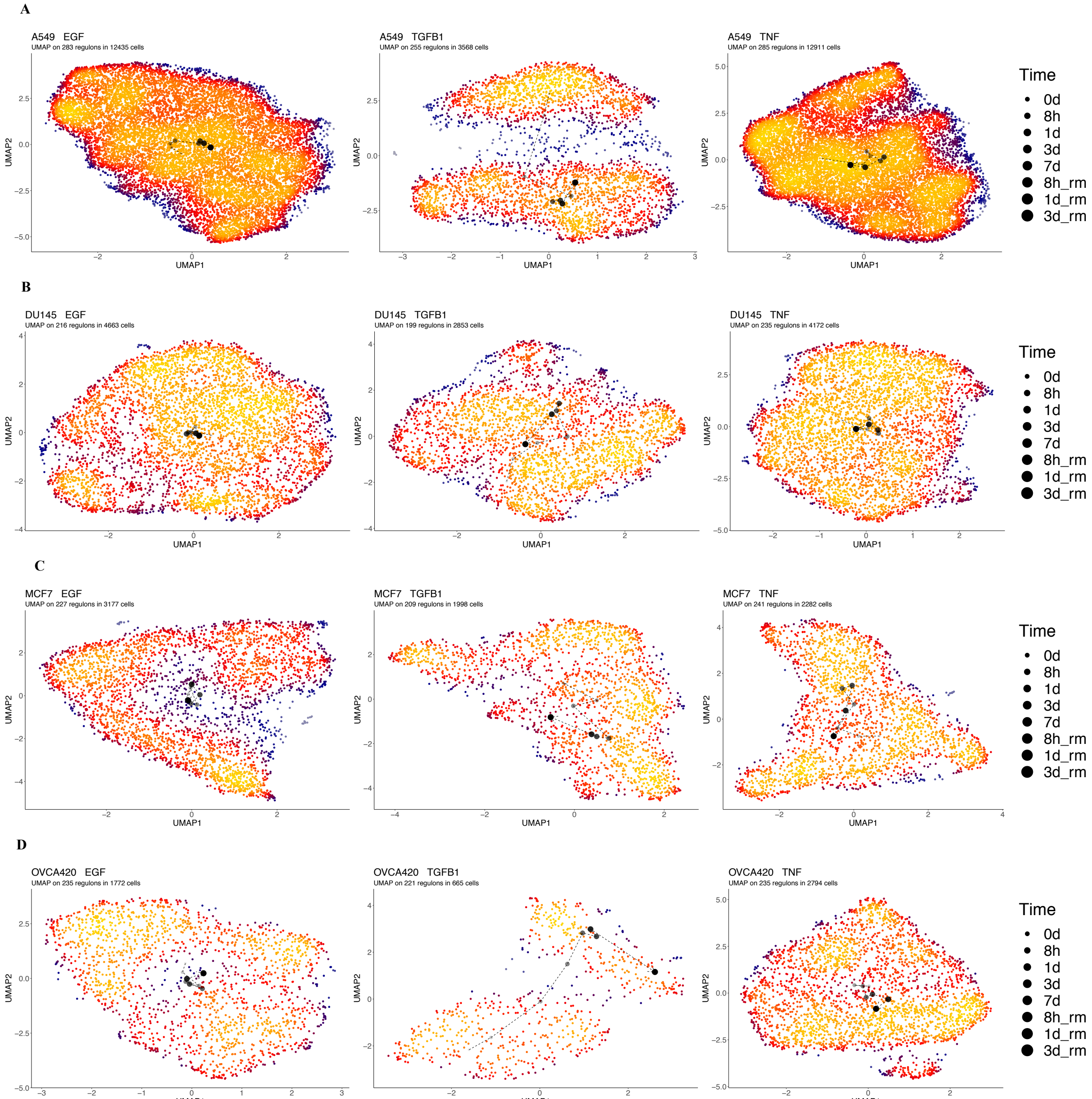

Figure S9. UMAP projections of regulon activity in **(A)** A549, **(B)** DU145, **(C)** MCF7, and **(D)** OVCA420 cells. Overlaid black points show centroids for each timepoint. Timepoint is also indicated by point size and transparency, and centroids for each timepoint are connected with a dashed line.

Table S1. Shared TFs in the core GRC, their phenotypic state assignments, and references.

Gene	State	Evidence (SI Reference Number)
BHLHE40	M?	1,31,40
CEBPB	M	2,19
CREB3	M	39
CREM		7,16
ELK3	M	5
ESRRA		37
ETS1	M	17
ETS2	M	3
FOS	E	22
FOSB	M	23,24
FOSL1	M	15
IRF1	M?	13,43,44
IRF2		
IRF3	E	42
IRF7	M	43
JUN		32
JUNB		32
KLF6	M	10
MAFG	E?	12
MYC	M	6
NFE2L3	M	45
NFKB	M	11,18,27
RELB	M	20
SOX4	M	38
SPDEF	E	35
STAT1		
STAT3	M	21
XBP1	M	34

Table S2. Literature-based core network interactions and supporting evidence.

Source	Target	Interaction Type*	Description	SI Reference Number
Sig	NFKB	1	TGFB/TNF/EGR activates NFKB	30, 36, 41
Sig	AP1	1	TGFB/TNF/EGR activates JUN/FOS	4,30,41
NFKB	AP1	1	NFKB activates FOS	8
AP1	AP1	2	JunB inhibits FOS	32
NFKB	NFKB	2	NFKB IKB form negative feedback loop	14,28
NFKB	SNAIL	1	NKFB signaling stablize SNAIL	41
SNAIL	SNAIL	2	SNAIL binds its own promoter and controls its expression	26
AP1	SNAIL	1	AP1 induces SNAIL expression	20
SNAIL	ZEB	1	SNAIL indirectly activates ZEB	25
ZEB	ZEB	1	ZEB indirectly activates ZEB expression	9,29
SNAIL	CDH1	2	SNAIL suppresses CDH1	33
ZEB	CDH1	2	SNAIL suppresses CDH1	33

*Interaction type 1 denotes activation and type 2 denotes inhibition.

Table S3. Properties of the networks shown in Fig. 6.

CellLine	A549			DU145			MCF7			OVCA420		
Signal	EGF	TGFB1	TNF	EGF	TGFB1	TNF	EGF	TGFB1	TNF	EGF	TGFB1	TNF
MiPositive	0.3	0.4	0.2	0.1	0.1	0.1	0.65	0.2	0.15	0.25	0.4	0.15
MiNegative	0.05	0.1	0.2	0.15	0.05	0.1	0.05	0.25	0.1	0.15	0.1	0.05
Accuracy	0.6285	0.9395	0.4095	0.955	0.5115	0.481	0.6625	0.529	0.8785	0.9845	0.877	0.0385
Nodes	30	13	33	46	39	46	30	40	60	31	29	49
Interactions	66	40	99	111	76	85	49	115	245	98	55	121
PositiveInteractions	18	36	96	109	70	83	2	111	233	96	34	101
EModels	977	1645	817	1769	983	948	800	1037	1730	1808	1407	8
MModels	280	234	2	141	40	14	525	21	27	161	347	69
Mgenes	12	12	22	22	24	29	2	5	35	16	16	4
Egenes	11	1	2	0	4	1	5	0	6	1	7	8
Transitivity	0.19466	0.49565	0.41758	0.21552	0.17045	0.15254	0.02013	0.26678	0.31181	0.37755	0.21145	0.21809
MeanDistance	9.03103	3.75000	8.08617	22.00000	18.31242	27.17343	20.52069	14.63397	17.95000	10.72258	15.96675	19.27806

SI References

1. H-J Cho; N Oh; J-H Park; K-S Kim; H-K Kim; E Lee; S Hwang; S-J Kim; K-S Park. ZEB1 Collaborates with ELK3 to Repress E-Cadherin Expression in Triple-Negative Breast Cancer Cells. *Mol Cancer Res* 17, 2257–2266 (2019)

2. L Hill; G Browne; E Tulchinsky. ZEB/miR-200 feedback loop: At the crossroads of signal transduction in cancer. *Int J Cancer* 132, 745–754 (2013)

3. Y Li; Y Liu; Y Xu; JJ Voorhees; GJ Fisher. UV irradiation induces Snail expression by AP-1 dependent mechanism in human skin keratinocytes. *Journal of Dermatological Science* 60, 105–113 (2010)

4. W Shi; Z Chen; L Li; H Liu; R Zhang; Q Cheng; D Xu; L Wu. Unravel the molecular mechanism of XBP1 in regulating the biology of cancer cells. *J Cancer* 10, 2035–2046 (2019)

5. C Li; Z Wang; Y Chen; M Zhou; H Zhang; R Chen; F Shi; C Wang; Z Rui. Transcriptional silencing of ETS-1 abrogates epithelial-mesenchymal transition resulting in reduced motility of pancreatic cancer cells. *Oncology Reports* 33, 559–565 (2015)

6. Y Wu; BP Zhou. TNF-α/NF-κB/Snail pathway in cancer cell migration and invasion. *Br J Cancer* 102, 639–644 (2010)

7. JJ Steffan; S Koul; RB Meacham; HK Koul. The Transcription Factor SPDEF Suppresses Prostate Tumor Metastasis. *J Biol Chem* 287, 29968–29978 (2012)

8. Y Wu; F Sato; T Yamada; UK Bhawal; T Kawamoto; K Fujimoto; M Noshiro; H Seino; S Morohashi; K Hakamada; Y Abiko; Y Kato; H Kijima. The BHLH transcription factor DEC1 plays an important role in the epithelial-mesenchymal transition of pancreatic cancer. *International Journal of Oncology* 41, 1337–1346 (2012)

9. SB Pakala; K Singh; SDN Reddy; K Ohshiro; D-Q Li; L Mishra; R Kumar. TGF-β1 signaling targets metastasis-associated protein 1, a new effector in epithelial cells. *Oncogene* 30, 2230–2241 (2011)

10. H Chang; Y Liu; M Xue; H Liu; S Du; L Zhang; P Wang. Synergistic action of master transcription factors controls epithelial-to-mesenchymal transition. *Nucleic Acids Res* 44, 2514–2527 (2016)

11. N Tiwari; VK Tiwari; L Waldmeier; PJ Balwierz; P Arnold; M Pachkov; N Meyer-Schaller; D Schübeler; E van Nimwegen; G Christofori. Sox4 Is a Master Regulator of Epithelial-Mesenchymal Transition by Controlling Ezh2 Expression and Epigenetic Reprogramming. *Cancer Cell* 23, 768–783 (2013)

12. S Peiro. Snail1 transcriptional repressor binds to its own promoter and controls its expression. *Nucleic Acids Research* 34, 2077–2084 (2006)

13. H Peinado; D Olmeda; A Cano. Snail, Zeb and bHLH factors in tumour progression: an alliance against the epithelial phenotype? *Nat Rev Cancer* 7, 415–428 (2007)

14. J Wouters; Z Kalender-Atak; L Minnoye; KI Spanier; M De Waegeneer; CB González-Blas; D Mauduit; K Davie; G Hulselmans; A Najem; M Dewaele; F Rambow; S Makhzami; V Christiaens; F Ceyssens; G Ghanem; J-C Marine; S Poovathingal; S Aerts. Single-cell gene regulatory network analysis reveals new melanoma cell states and transition trajectories during phenotype switching. Genomics (2019)

15. M-M Yu; Y-H Feng; L Zheng; J Zhang; G-H Luo. Short hairpin RNA-mediated knockdown of nuclear factor erythroid 2-like 3 exhibits tumor-suppressing effects in hepatocellular carcinoma cells. *WJG* 25, 1210–1223 (2019)

16. J Holian; W Qi; DJ Kelly; Y Zhang; E Mreich; CA Pollock; X-M Chen. Role of Krüppel-like factor 6 in transforming growth factor-β1-induced epithelial-mesenchymal transition of proximal tubule cells. *American Journal of Physiology-Renal Physiology* 295, F1388–F1396 (2008)
17. K Asanoma; G Liu; T Yamane; Y Miyanari; T Takao; H Yagi; T Ohgami; A Ichinoe; K Sonoda; N Wake; K Kato. Regulation of the Mechanism of *TWIST1* Transcription by BHLHE40 and BHLHE41 in Cancer Cells. *Mol Cell Biol* 35, 4096–4109 (2015)
18. SJ Serrano-Gomez; M Maziveyi; SK Alahari. Regulation of epithelial-mesenchymal transition through epigenetic and post-translational modifications. *Mol Cancer* 15, 18 (2016)
19. KB Cho; MK Cho; WY Lee; KW Kang. Overexpression of c-myc induces epithelial mesenchymal transition in mammary epithelial cells. *Cancer Letters* 293, 230–239 (2010)
20. DA Potoyan; PG Wolynes. On the dephasing of genetic oscillators. *Proc Natl Acad Sci USA* 111, 2391–2396 (2014)
21. A Jana; NL Krett; G Guzman; A Khalid; O Ozden; JJ Staudacher; J Bauer; SH Baik; T Carroll; C Yazici; B Jung. NFkB is essential for activin-induced colorectal cancer migration via upregulation of PI3K-MDM2 pathway. *Oncotarget* 8 (2017)
22. BRB Pires; AL Mencalha; GM Ferreira; WF de Souza; JA Morgado-Díaz; AM Maia; S Corrêa; ESFW Abdelhay. NF-kappaB Is Involved in the Regulation of EMT Genes in Breast Cancer Cells. *PLoS ONE* 12, e0169622 (2017)
23. S Fujioka; J Niu; C Schmidt; GM Sclabas; B Peng; T Uwagawa; Z Li; DB Evans; JL Abbruzzese; PJ Chiao. NF- B and AP-1 Connection: Mechanism of NF- B-Dependent Regulation of AP-1 Activity. *Molecular and Cellular Biology* 24, 7806–7819 (2004)
24. L Jiang; J Liu; Y Shi; B Tang; T He; J Liu; J Fan; B Wu; X Xu; Y Zhao; F Qian; Y Cui; P Yu. MTMR2 promotes invasion and metastasis of gastric cancer via inactivating IFNγ/STAT1 signaling. *J Exp Clin Cancer Res* 38, 206 (2019)

25. M Yu; H Xue; Y Wang; Q Shen; Q Jiang; X Zhang; K Li; M Jia; J Jia; J Xu; Y Tian. miR-345 inhibits tumor metastasis and EMT by targeting IRF1-mediated mTOR/STAT3/AKT pathway in hepatocellular carcinoma. *International Journal of Oncology* 50, 975–983 (2017)

26. YC Jia; JY Wang; YY Liu; B Li; H Guo; AM Zang. LncRNA MAFG-AS1 facilitates the migration and invasion of NSCLC cell via sponging miR-339-5p from MMP15. *Cell Biol Int* 43, 384–393 (2019)

27. J Schütte; J Viallet; M Nau; S Segal; J Fedorko; J Minna. jun-B inhibits and c-fos stimulates the transforming and trans-activating activities of c-jun. *Cell* 59, 987–997 (1989)

28. JD Kearns; S Basak; SL Werner; CS Huang; A Hoffmann. IκBε provides negative feedback to control NF-κB oscillations, signaling dynamics, and inflammatory gene expression. *The Journal of Cell Biology* 173, 659–664 (2006)

29. P Xu; S Bailey-Bucktrout; Y Xi; D Xu; D Du; Q Zhang; W Xiang; J Liu; A Melton; D Sheppard; HA Chapman; JA Bluestone; R Derynck. Innate Antiviral Host Defense Attenuates TGF-β Function through IRF3-Mediated Suppression of Smad Signaling. *Molecular Cell* 56, 723–737 (2014)

30. AK Kiemer; K Takeuchi; MP Quinlan. Identification of genes involved in epithelial-mesenchymal transition and tumor progression. *Oncogene* 20, 6679–6688 (2001)

31. C Tiraby; BC Hazen; ML Gantner; A Kralli. Estrogen-Related Receptor Gamma Promotes Mesenchymal-to-Epithelial Transition and Suppresses Breast Tumor Growth. *Cancer Research* 71, 2518–2528 (2011)

32. M Romagnoli; K Belguise; Z Yu; X Wang; E Landesman-Bollag; DC Seldin; D Chalbos; S Barillé-Nion; P Jézéquel; ML Seldin; GE Sonenshein. Epithelial-to-Mesenchymal Transition Induced by TGF-β1 Is Mediated by Blimp-1–Dependent Repression of BMP-5. *Cancer Res* 72, 6268–6278 (2012)

33. C-W Li; W Xia; L Huo; S-O Lim; Y Wu; JL Hsu; C-H Chao; H Yamaguchi; N-K Yang; Q Ding; Y Wang; Y-J Lai; AM LaBaff; T-J Wu; B-R Lin; M-H Yang; GN Hortobagyi; M-C Hung. Epithelial-Mesenchymal Transition Induced by TNF- Requires NF- B-Mediated Transcriptional Upregulation of Twist1. *Cancer Research* 72, 1290–1300 (2012)
34. J Yang; B Tian; H Sun; RP Garofalo; AR Brasier. Epigenetic silencing of IRF1 dysregulates type III interferon responses to respiratory virus infection in epithelial to mesenchymal transition. *Nat Microbiol* 2, 17086 (2017)
35. H-W Lo; S-C Hsu; W Xia; X Cao; J-Y Shih; Y Wei; JL Abbruzzese; GN Hortobagyi; M-C Hung. Epidermal Growth Factor Receptor Cooperates with Signal Transducer and Activator of Transcription 3 to Induce Epithelial-Mesenchymal Transition in Cancer Cells via Up-regulation of *TWIST* Gene Expression. *Cancer Res* 67, 9066–9076 (2007)
36. D Chen; JS Davis. Epidermal growth factor induces c-fos and c-jun mRNA via Raf-1/MEK1/ERK-dependent and -independent pathways in bovine luteal cells. *Molecular and Cellular Endocrinology* 200, 141–154 (2003)
37. L Sun; G Carpenter. Epidermal growth factor activation of NF-κB is mediated through IκBα degradation and intracellular free calcium. *Oncogene* 16, 2095–2102 (1998)
38. J Li; F Shan; G Xiong; X Chen; X Guan; J-M Wang; W-L Wang; X Xu; Y Bai. EGF-induced C/EBP participates in EMT by decreasing the expression of miR-203 in esophageal squamous cell carcinoma cells. *Journal of Cell Science* 127, 3735–3744 (2014)
39. F Sato; UK Bhawal; T Yoshimura; Y Muragaki. DEC1 and DEC2 Crosstalk between Circadian Rhythm and Tumor Progression. *J Cancer* 7, 153–159 (2016)
40. S Mahner; C Baasch; J Schwarz; S Hein; L Wölber; F Jänicke; K Milde-Langosch. C-Fos expression is a molecular predictor of progression and survival in epithelial ovarian carcinoma. *Br J Cancer* 99, 1269–1275 (2008)

41. R Kessler; A Zacharova-Albinger; NB Laursen; M Kalousek; R Klemenz. Attenuated expression of the serum responsive T1 gene in ras transformed fibroblasts due to the inhibition of c-fos gene activity. *Oncogene* 18, 1733–1744 (1999)
42. B-T Preca; K Bajdak; K Mock; W Lehmann; V Sundararajan; P Bronsert; A Matzge-Ogi; V Orian-Rousseau; S Brabletz; T Brabletz; J Maurer; MP Stemmler. A novel ZEB1/HAS2 positive feedback loop promotes EMT in breast cancer. *Oncotarget* 8 (2017)
43. C Bornstein; D Winter; Z Barnett-Itzhaki; E David; S Kadri; M Garber; I Amit. A Negative Feedback Loop of Transcription Factors Specifies Alternative Dendritic Cell Chromatin States. *Molecular Cell* 56, 749–762 (2014)
44. Y Nakabeppu; D Nathans. A naturally occurring truncated form of FosB that inhibits Fos/Jun transcriptional activity. *Cell* 64, 751–759 (1991)
45. GM Fimia; D De Cesare; P Sassone-Corsi. A Family of LIM-Only Transcriptional Coactivators: Tissue-Specific Expression and Selective Activation of CREB and CREM. *Molecular and Cellular Biology* 20, 8613–8622 (2000)
